# The small heat shock protein 20 RSI2 interacts with and is required for stability and function of tomato resistance protein I-2

**DOI:** 10.1111/j.1365-313X.2010.04260.x

**Published:** 2010-06-16

**Authors:** Gerben van Ooijen, Ewa Lukasik, Harrold A van den Burg, Jack H Vossen, Ben J C Cornelissen, Frank L W Takken

**Affiliations:** Department of Plant Pathology, Swammerdam Institute for Life Sciences, University of AmsterdamScience Park 904, 1098 XH Amsterdam, The Netherlands

**Keywords:** HSP20, NB-LRR protein, alpha crystallin domain, hypersensitive response, resistasome, immunity

## Abstract

Race-specific disease resistance in plants depends on the presence of resistance (*R*) genes. Most *R* genes encode NB-ARC-LRR proteins that carry a C-terminal leucine-rich repeat (LRR). Of the few proteins found to interact with the LRR domain, most have proposed (co)chaperone activity. Here, we report the identification of RSI2 (Required for Stability of I-2) as a protein that interacts with the LRR domain of the tomato R protein I-2. RSI2 belongs to the family of small heat shock proteins (sHSPs or HSP20s). HSP20s are ATP-independent chaperones that form oligomeric complexes with client proteins to prevent unfolding and subsequent aggregation. Silencing of RSI2-related HSP20s in *Nicotiana benthamiana* compromised the hypersensitive response that is normally induced by auto-active variants of I-2 and Mi-1, a second tomato R protein. As many HSP20s have chaperone properties, the involvement of RSI2 and other R protein (co)chaperones in I-2 and Mi-1 protein stability was examined. RSI2 silencing compromised the accumulation of full-length I-2 *in planta*, but did not affect Mi-1 levels. Silencing of heat shock protein 90 (HSP90) and SGT1 led to an almost complete loss of full-length I-2 accumulation and a reduction in Mi-1 protein levels. In contrast to SGT1 and HSP90, RSI2 silencing led to accumulation of I-2 breakdown products. This difference suggests that RSI2 and HSP90/SGT1 chaperone the I-2 protein using different molecular mechanisms. We conclude that I-2 protein function requires RSI2, either through direct interaction with, and stabilization of I-2 protein or by affecting signalling components involved in initiation of the hypersensitive response.

## Introduction

Resistance (R) proteins in plants mediate recognition of specific pathogen-derived factors called Avirulence (Avr) proteins. Upon Avr perception, R proteins initiate defence responses that limit further pathogen ingress. These responses often result in macroscopically visible cell death, referred to as the hypersensitive response (HR).

The majority of R proteins are NB-ARC-LRR proteins, which contain a central nucleotide-binding and -hydrolysing domain (NB-ARC) and a C-terminal leucine-rich repeat (LRR) domain (Martin *et al.*, 2003; van Ooijen *et al.*, 2007; Tameling and Takken, 2008). The LRR mediates both intra- and intermolecular interactions (reviewed by Lukasik and Takken, 2009). Known intermolecular interactions include those with (co)chaperones, with the best studied being heat shock protein 90 (HSP90) that physically interacts with the LRRs of the R proteins I-2, RPM1, N and Rx (Hubert *et al.*, 2003; Lu *et al.*, 2003; Liu *et al.*, 2004; de la Fuente van Bentem *et al.*, 2005). HSP90 is a highly conserved ATP-dependent molecular chaperone that is responsible for the stability and function of a large number of proteins, collectively referred to as HSP90 client proteins (Pearl and Prodromou, 2006). The activity of HSP90 is regulated by interactions with co-chaperones (Pearl and Prodromou, 2006). Some of these co-chaperones also directly interact with HSP90 client proteins. For example, the co-chaperone protein phosphatase 5 (PP5) binds both the C-terminus of HSP90 and the LRR domain of R protein I-2 (de la Fuente van Bentem *et al.*, 2005). Another HSP90-interacting co-chaperone, SGT1 (suppressor of the G2 allele of Skp1), has been shown to interact with the LRR of the barley resistance protein Mla-1 (Bieri *et al.*, 2004). HSP90 and SGT1 have also been shown to interact with a third partner; RAR1 (Required for Mla12 Resistance) (Shirasu *et al.*, 1999; Hubert *et al.*, 2003; Takahashi *et al.*, 2003; Bieri *et al.*, 2004; Liu *et al.*, 2004; Azevedo *et al.*, 2006; Boter *et al.*, 2007; Heise *et al.*, 2007). The combined activities of the three (co)chaperones RAR1, SGT1 and HSP90 has been shown to be important for R protein stability and accumulation, and thus for R protein-mediated signalling responses (Zhang *et al.*, 2004; Azevedo *et al.*, 2006; Boter *et al.*, 2007).

Another class of proteins with chaperone-associated functions are small heat shock proteins (sHSPs), or HSP20s, which range in size from 12 to 43 kDa. HSP20s are of variable sequence, and are characterized by a conserved domain of approximately 90 residues forming an α-crystallin domain (ACD) (Caspers *et al.*, 1995). These proteins form large oligomers and perform their ATP-independent chaperone function *in vitro* by binding to (partially) denatured proteins (Lee *et al.*, 1995; Helm *et al.*, 1997; Kirschner *et al.*, 2000). *In vivo*, HSP20s are believed to confer a protective function by preventing unfolding or disassembly of other proteins (van Montfort *et al.*, 2001a). HSP20s probably maintain denatured proteins in a folding-competent state to allow subsequent ATP-dependent disaggregation by the HSP70/90 chaperone system (Kotak *et al.*, 2007; Liberek *et al.*, 2008).

Here, we describe the identification of RSI2 (Required for Stability of I-2), an HSP20 member that specifically interacts with the tomato (*Solanum lycopersicum*) R protein I-2. I-2 confers resistance to *Fusarium oxysporum* (Simons *et al.*, 1998). In addition to analysing the physical interaction between I-2 and RSI2, we used a virus-induced gene silencing approach to analyse the functional involvement of RSI2 and other known (co)chaperones in the HR mediated by auto-active variants of I-2 and a second tomato R protein, Mi-1. Mi-1 belongs to a different subgroup of NB-ARC-LRR proteins (van Ooijen *et al.*, 2007), and confers resistance to root-knot nematodes (*Meloidogyne* sp.), potato top aphid (*Macrosiphum euphorbiae*) and whitefly (*Bemisia tabaci*) (Vos *et al.*, 1998). Furthermore, we analysed the effect of silencing of RSI2 and other (co)chaperones on I-2 and Mi-1 protein abundance and stability *in planta.*

## Results

### RSI2 interacts with the I-2 LRR domain in the yeast two-hybrid system

**Figure 1:**
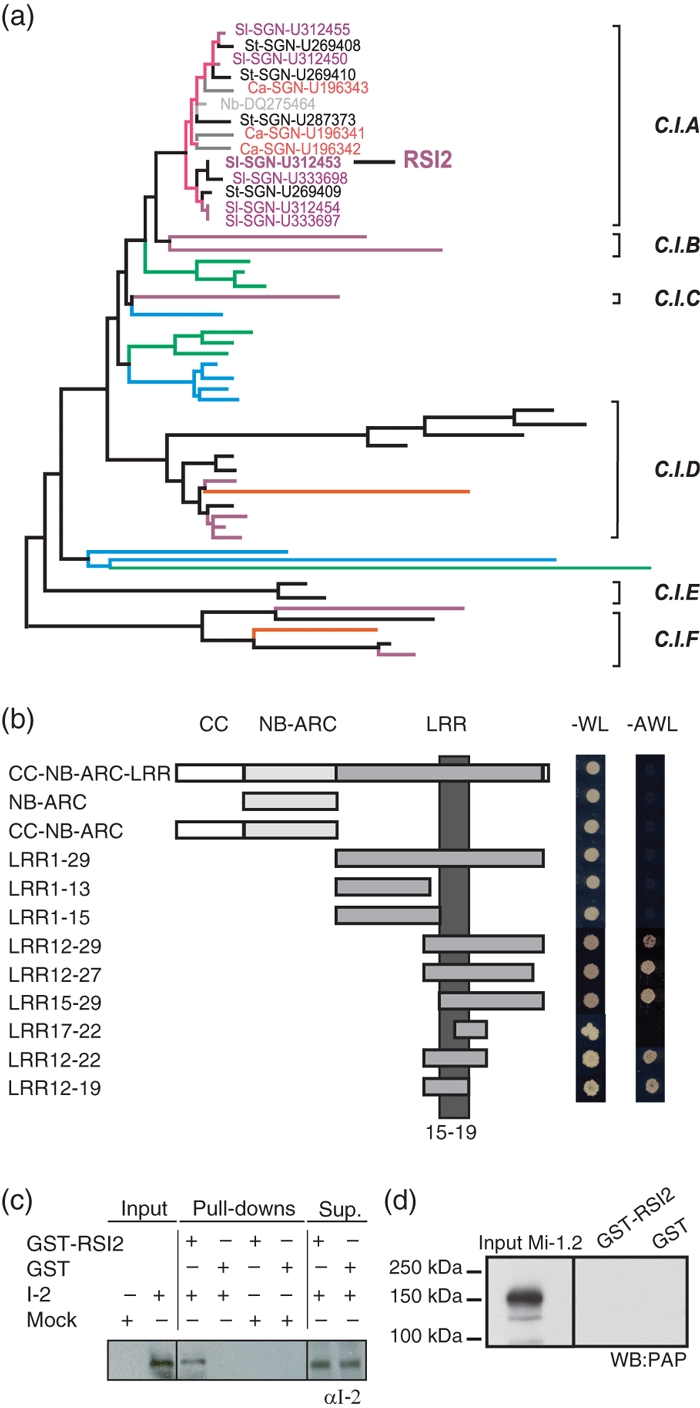
RSI2 interacts with I-2.(a) Evolutionary relationship of class I (C.I.) cytosolic HSP20s from tomato (Sl, purple), potato (St, black), *Capsicum annuum* (Ca, red) and *N. benthamiana* (Nb, grey). Six sub-clades can be distinguished (C.I.A to C.I.F). The tree contains 51 taxa, and is part of a larger tree containing 113 taxa (Figure S1).(b) Yeast two-hybrid assays showing interactions between RSI2 and truncated versions of I-2. The presence of bait and prey plasmids was confirmed by growth on minimal medium lacking tryptophan and leucine (–WL), and the interaction between bait and prey proteins was analysed by growth on minimal medium lacking tryptophan, leucine and adenine (–AWL). The dark grey bar highlights the I-2 region required for RSI2 interaction.(c) Western blot on total protein lysates from *N. benthamiana* leaves transiently expressing I-2 or mock-infiltrated and probed with I-2 antibody (αI-2). The presence of full-length R proteins in these extracts is shown in the left lanes. GST–RSI2 and GST proteins immobilized on glutathione Sepharose beads were incubated with these extracts as indicated (±). GST?–RSI2 and GST interacting proteins were subjected to SDS–PAGE and Western blot analysis to detect the presence of I-2. The supernatant remaining after pull-down was blotted to show that I-2 stability was unaffected by the various experimental conditions.(d) As (c), but using TAP-tagged Mi-1 and the PAP antibody to detect TAP-tagged Mi-1.

We reported previously the identification of RSI2 (originally referred to as an HSP17 protein) and PP5 as interactors of the I-2 LRR domain in a yeast two-hybrid screen (de la Fuente van Bentem *et al.*, 2005). PP5 was identified using LRR1–29 as bait, whereas RSI2 was identified using a different bait, LRR12–29, which corresponds to amino acid residues 823–1250 (LRR annotation as described by de la Fuente van Bentem *et al.*, 2005). Screening of 6 × 10^6^ clones of a tomato cDNA library with bait LRR12–29 revealed two independent interacting clones. The two cDNA clones carried 733 and 742 bp inserts (Genbank accession number AY150040). The inserts are overlapping and differ only in the length of their 5′ UTRs. They encode a full-length HSP20, based on the presence of an α-crystallin domain (ACD) fused to an N-terminal domain, with a predicted mass of 17.8 kDa. Plants contain at least six subclasses of HSP20 proteins targeted to various cellular compartments (Waters and Vierling, 1999; Scharf *et al.*, 2001). A phylogenetic tree derived from multiple sequence alignment of the ACDs of tomato, potato, pepper, rice and Arabidopsis HSP20 sequences clearly shows the various subfamilies (classification as described by Scharf *et al.*, 2001), and places RSI2 in class I of the cytosolic HSP20s (Figure 1a, Figure S1 and Appendix S1). The structure of the class I wheat HSP20 (van Montfort *et al.*, 2001a) reveals that the ACD adopts a β-sandwich fold consisting of two layers of five β-sheets flanked by helices and loops. A similar fold is predicted to be present in other HSP20s. Strikingly, we noted a strong paired-clustering of paralogues over orthologues for cytosolic class I proteins, and paralogues appear to have expanded in Solanaceae. This is suggestive of a rapid birth and death process, implying that this class of HSP20s (including RSI2) is under strong selection in the various hosts. Other classes of HSP20s targeted to other cellular compartments (e.g. mitochondria, peroxisomes, etc.) appear to have undergone less diversification.

To assess the specificity of the I-2/HSP20 interaction, representative ACD members of class I were selected based on the phylogenetic tree (Figure S1). Full-length cDNAs were amplified from tomato EST sequences provided by the Kazusa DNA Research Institute (Kisarazu, Chiba, Japan). Two closely related homologues from class IA were selected (SL-SGN-U312450 and SL-SGN-U312454). One EST (SL-SGN-U316206) was also selected from class ID to represent a more distantly related homologue. The interaction of these homologues with I-2 LRR12–29 was analysed using yeast two-hybrid assays, and accumulation of the HSP20 proteins in yeast was verified by Western blot analysis (Figure S2b). Of the four homologues analysed, RSI2 was the only HSP20 that interacted with I-2 LRR12–29 (Figure S2a), which implies that the interaction between I-2 and RSI2 is specific.

To pinpoint the region of the I-2 protein responsible for the interaction with RSI2, various N- and C-terminal truncations of the I-2 protein were analysed for their interaction with RSI2 in yeast two-hybrid assays (Figure 1b). The minimal RSI2-interacting region of the I-2 LRR domain lies within LRR15–19, corresponding to amino acids 906–1015 (Figure 1b). Notably, the full-length I-2 protein and the full-length LRR domain (LRR1–29) did not interact with RSI2 when expressed in yeast (de la Fuente van Bentem *et al.*, 2005).

### GST–RSI2 fusion protein interacts with I-2 from plant protein extracts

We next investigated whether recombinant RSI2 associates with plant-produced I-2 in plant protein extracts. We performed pull-down experiments using *Escherichia coli*-produced GST–RSI2 and GST alone with non-tagged plant-produced I-2 rather than immunoprecipitation of proteins produced *in planta*, as this approach has previously been used successfully to reveal interactions between other members of an I-2 complex in plant protein extracts (de la Fuente van Bentem *et al.*, 2005). The two GST bait proteins were affinity-purified and subsequently analysed by Coomassie staining of an SDS–PAGE gel. As shown in Figure S3, the proteins migrated according to their expected molecular weights (44 and 26 kDa), and little contamination of co-purifying protein was observed. Endogenous I-2 is expressed at very low levels in tomato and was undetectable in total protein leaf extracts using our affinity-purified I-2 antibody (Tameling *et al.*, 2002), and labelling of I-2 with a peptide tag abolishes its biological activity (van Ooijen *et al.*, 2008b). To achieve detectable expression levels, we produced full-length I-2 by transient transformation of *Nicotiana benthamiana* leaves using agroinfiltration. The *N. benthamiana*-produced full-length I-2 protein was readily detected in I-2-transformed plants, but not in mock-infiltrated plants (Figure 1c). Next, total protein extracts of *N. benthamiana* leaves infiltrated with either *A. tumefaciens* carrying I-2 constructs or with buffer were incubated with beads loaded with GST or GST–RSI2. The stability of the I-2 protein during the assay did not differ between the GST and GST–RSI2 samples, as shown by Western blot analysis of the supernatant fractions after GST pull-down. Moreover, I-2 was consistently co-purified with GST–RSI2, but not with the control containing GST alone (Figure 1c). The specific co-precipitation of I-2 with GST–RSI2 indicates that RSI2 interacts with the I-2 protein complex present in plant extracts.

We analysed the interaction of GST–RSI2 with the R protein Mi-1 in a similar manner. A tandem affinity purification (TAP)-tagged version of Mi-1 was used, as the polyclonal Mi-1 antibody is known to cross-react with the GST tag (van Ooijen *et al.*, 2008b). The TAP tag does not appear to affect Mi-1 protein function, as the TAP-tagged auto-active mutant Mi-1^H840A^ (van Ooijen *et al.*, 2008b) induces an HR comparable to that of the non-tagged auto-active mutant in *N. benthamiana* (Figure S4). Full-length TAP-tagged Mi-1 can be readily detected on Western blot using PAP (peroxidise anti-peroxidase) antibody (Figure 1d). However, TAP-tagged Mi-1 did not co-precipitate with the GST–RSI2 fusion protein under the conditions used. These results indicate that, although RSI2 and I-2 can form a complex, RSI2 does not interact with the Mi-1 protein complex under the same conditions.

### VIGS reveals a role for RSI2 in HR mediated by I-2 and Mi-1

**Figure 2:**
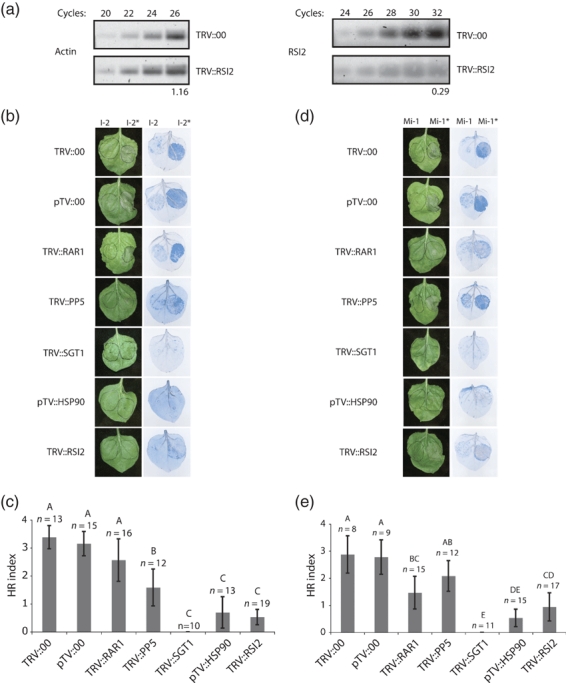
VIGS reveals a role for RSI2 in I-2- and Mi-1-mediated HR signaling.(a) Silencing efficiency of *RSI2* was determined using semi-quantitative RT-PCR (right panel). Actin expression levels were measured as a control for equal cDNA quantity and quality (left panel). The number of PCR cycles is indicated at the top. The relative signal intensity compared to TRV::00 (indexed at 1) is indicated below.(b, d) Agroinfiltration of wild-type (left side of the leaf) or constitutively active mutants (right side of the leaf) of I-2 (b) and Mi-1 (d) into *N. benthamiana* 3 weeks after induction of silencing using the indicated TRV vectors. Photographs were taken 3 days after agroinfiltration, and representative leaves were stained using trypan blue to visualize cell death (right panels).(c, e) Severity of the HR upon I-2^D495V^ (c) or Mi-1^D841V^ (e) expression in silenced plants. HR was quantified visually on a scale from 0 (no symptoms) to 4 (full necrosis). Significant differences were determined by one-way anova (*P* < 0.05) and are indicated by different letters above the bars. The error bars represent the 95% confidence level; *n* is the number of plants analysed.

R protein function depends on the activities of a number of chaperones or chaperone-associated proteins (de la Fuente van Bentem *et al.*, 2005; Boter *et al.*, 2007). To analyse whether RSI2 is also required for R protein function, we used virus-induced gene silencing (VIGS) of *RSI2*. For comparison, the (co)chaperones SGT1, RAR1, PP5 and HSP90 were included in these experiments. VIGS was induced using the tobacco rattle virus (TRV) silencing system, which was delivered by agroinfiltration of viral vector constructs into the leaflets of 2-week-old *N. benthamiana* plants (Ratcliff *et al.*, 2001). To confirm onset and spread of silencing over time, phytoene desaturase (PDS) was used as a visible marker (Demmig-Adams and Adams, 1992). The nearly complete photobleaching that was consistently observed 3 weeks after agroinfiltration indicates extensive *PDS* silencing throughout these plants at this time point (Figure S5). To assess *RSI2* silencing levels, specific primers were designed on the basis of the closest *N. benthamiana* homologue (shown in Figure 1b) present in the Institute for Genomic Research (TIGR) database (GenBank accession number DQ275464). In the sequenced region, this *N. benthamiana* gene is only 80% identical to *RSI2*, and is not predicted to be a silencing target (Xu *et al.*, 2006). However, the relative expression level of this *RSI2* homologue was reduced to 30% compared to the non-silenced controls in leaf extracts (Figure 2a). The presence of other, possibly even more closely related, unknown *RSI2* genes in the *N. benthamiana* genome cannot be excluded, and silencing might therefore also target other genes encoding RSI2 homologues belonging to class IA. Efforts to analyse total class I protein levels by Western blotting of silenced plants were not successful because the affinity of several tested antibodies was insufficient to detect HSP20s in *N. benthamiana* protein extracts.

To assess I-2 function in the silenced plants, leaves were agroinfiltrated with constructs expressing wild-type I-2 (left side of the leaves) and the auto-active I-2^D495V^ (I-2*) mutant (right side of the leaves) (Figure 2b,c). The level of cell death induced by I-2^D495V^ was scored 3 days after agroinfiltration on a scale from 0 (absolutely no tissue collapse) to 4 (fully developed HR in the entire infiltrated region). The scale is shown in Figure S6. To enhance clarity of the HR, the infiltrated leaves were stained for cell death using trypan blue (Figure 2b).

Leaves of plants infected with the empty virus control showed a clear HR upon expression of I-2^D495V^, but not upon expression of wild-type I-2 (Figure 2b). This result showed that induction of the HR by I-2^D495V^ was not compromised by infection with TRV. Silencing of the genes encoding the established R protein (co)chaperones HSP90 or SGT1 severely suppressed the HR triggered by I-2^D495V^. Similarly, no or only minor tissue collapse induced by I-2^D495V^ was observed in the *RSI2* silenced plants (0.5 ± 0.3) compared to control plants (3.4 ± 0.4) (Figure 2c). A one-way anova showed that the severity of HR symptoms in *RSI2*, *HSP90* and *SGT1* silenced plants was significantly reduced, reaching similar low levels, indicating a requirement for each of these genes for I-2-mediated HR. Silencing of *PP5* only partially compromised I-2^D495V^-mediated HR, and *RAR1* silencing did not significantly affect the HR (Figure 2b,c).

To determine whether RSI2 is also involved in the HR mediated by Mi-1, we assessed induction of the HR in silenced plants by the auto-active mutant Mi-1^D841V^ (Mi-1*) (van Ooijen *et al.*, 2008b). In plants infected with empty virus, a strong HR (2.9 ± 0.7) was induced by this mutant (Figure 2d, right side of the leaves) but not by the wild-type Mi-1 protein (left side of the leaves). In plants silenced for *RSI2*, the HR induced by Mi-1^D841V^ was consistently and strongly compromised (0.9 ± 0.5) (Figure 2d,e). As with I-2^D495^, Mi-1^D841V^-mediated HR induction is also severely compromised upon *SGT1* and *HSP90* silencing. *PP5* and *RAR1* silencing also affected HR induction by Mi-1^D841V^, but to a lesser extent. These data conclusively show that the HR mediated by auto-active I-2 and Mi-1 is strongly reduced when expression of class IA *HSP20s* is diminished by gene silencing.

### *RSI2* silencing negatively affects I-2 protein accumulation

The suppressive effect of *SGT1* gene silencing on *N and Rx* function is a result of compromised R protein stability, possibly due to reduced chaperone activity (Azevedo *et al.*, 2006; Mestre and Baulcombe, 2006; Boter *et al.*, 2007). HSP20s are also associated with chaperone functions *in vitro* (Nover and Scharf, 1997; Kirschner *et al.*, 2000). To analyse whether the observed effects on the HR by silencing *RSI2*, *SGT1*, *HSP90*, *PP5* or *RAR1* can be attributed to reduced R protein stability, accumulation of I-2 and Mi-1 was analysed in the silenced plants.

**Figure 3:**
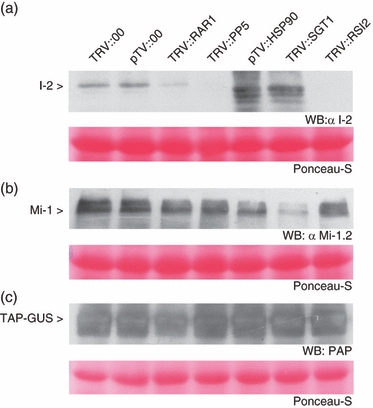
Expression levels of I-2, Mi-1 and GUS protein in silenced *Nicotiana benthamiana* leaves.*N. benthamiana* plants were silenced using the indicated TRV-based silencing constructs. Three weeks after induction of silencing, the upper leaves were agroinfiltrated with constructs expressing either I-2, Mi-1 or TAP-tagged GUS. One day after agroinfiltration, leaves were harvested and protein was extracted. Total protein (50 μg for I-2 and Mi-1 or 10 μg for TAP-tagged GUS) was loaded onto SDS–PAGE gels. Expression of I-2 (a), Mi-1 (b) or TAP-tagged GUS (c) was analysed by Western blotting using the antibody indicated. Equal loading was confirmed by Ponceau S staining of Rubisco (lower panels).

To exclude the possibility that silencing of any of these genes adversely influences transgene expression via agroinfiltration *per se*, we agroinfiltrated a construct expressing TAP-tagged GUS protein. Expression levels of this control protein were not altered by silencing any of the indicated genes (Figure 3c), as shown by Western blotting using an antibody recognising the TAP tag.

R protein accumulation in the silenced plants was assessed after agroinfiltration with I-2 and Mi-1 constructs. Compared to the vector controls, silencing of *RAR1* and *PP5* appeared to reduce I-2 protein levels (Figure 3a). Full-length I-2 protein was not detected at all in plants silenced for *SGT1* and *HSP90*. However, strong accumulation of smaller proteins reacting with the I-2 antibody (putative I-2-derived degradation products) was observed. Silencing of *RSI2* also abolished the accumulation of full-length I-2; however, this did not result in accumulation of smaller proteins that react with the I-2 antibody.

Mi-1 protein levels in *RSI2*, *RAR1* or *PP5* silenced plants were similar to those observed in empty virus controls (Figure 3b). Silencing of *SGT1* led to a clear reduction of Mi-1 protein abundance, whereas *HSP90* silencing only slightly affected Mi-1 protein accumulation. In neither case did we detect lower-molecular-weight products cross-reacting with the Mi-1 antibody. These data show that, although RSI2 is necessary for HR induction by auto-active Mi-1, silencing of *RSI2* does not directly affect accumulation of the Mi-1 protein.

In conclusion, our data indicate that HSP90 and SGT1 are both required to maintain I-2 and Mi-1 integrity and stability. The functional involvement of RSI2 in *I-2*-mediated HR is associated with I-2 protein stabilization, but no effect on Mi-1 stability was observed.

## Discussion

We here identify RSI2 as component of the I-2 protein complex, and show its requirement for the I-2- and Mi-1-mediated HR. We find that silencing of *RSI2* severely reduces I-2 protein accumulation *in planta* (Figure 3), indicating a role for RSI2 in chaperoning I-2. As shown in Figure 1(a), RSI2 is a class I cytosolic HSP20 protein. Members of this class of HSP20s have been described to form large assemblies that prevent aggregation of unfolded client proteins (Lee *et al.*, 1997; Lee and Vierling, 2000). HSP20 binding maintains these client proteins in a folding-competent state, allowing refolding by the ATP-dependent HSP70/HSP90 machinery (reviewed by Liberek *et al.*, 2008). In the absence of chaperone activity, a client protein will aggregate and be degraded.

We also analysed the effect on I-2- and Mi-1-mediated HR after silencing other (co)chaperones (SGT1, HSP90, RAR1 and PP5) that interact with R proteins. In agreement with a previous study, silencing of *SGT1*, *HSP90* or *RAR1* reduced I-2-mediated HR. In that study, silencing of *PP5* had no effect on I-2-mediated HR (de la Fuente van Bentem *et al.*, 2005), whereas a relatively small, but significant, suppression of I-2-mediated HR was found in the present study. The difference between these studies might be due to enhanced efficiency of the *Agrobacterium*-mediated transformation procedure and higher silencing levels in our study due to use of the enhanced transformation procedures described by Fu *et al.*, 2006. This is supported by the more extensive bleaching observed upon *PDS* silencing in our study (Figure S5) compared to the previous one (de la Fuente van Bentem *et al.*, 2005).

We found that the HR mediated by an auto-active variant of Mi-1 is dependent on SGT1 and HSP90, which is consistent with the involvement of these proteins in Mi-1-mediated resistance towards whitefly (Bhattarai *et al.*, 2007). RAR1 was found not to be involved in Mi-1-mediated whitefly resistance (Bhattarai *et al.*, 2007), but we found that this protein is required for full HR induction by Mi-1. This difference may suggest that the HR is not required for whitefly resistance.

Class I HSP20s (including RSI2) are proposed to act sequentially to HSP90 to disaggregate misfolded client proteins (Liberek *et al.*, 2008). Such a chaperone function for RSI2, i.e. maintaining misfolded I-2 in a soluble form to allow its refolding, agrees with the observed reduced accumulation of the full-length I-2 protein upon *RSI2* or *HSP90* silencing. Alternatively, RSI2 may function in direct conjunction with HSP90. The crystal structure of wheat HSP20 (van Montfort *et al.*, 2001b) reveals that its fold closely resembles that of the CS (CHORD and SGT1) domain (Finn *et al.*, 2006) found in SGT1 and p23, which are required for binding to HSP90 (Boter *et al.*, 2007). p23 regulates human HSP90 activity by direct interaction with the ATP-bound active form of HSP90 (Johnson *et al.*, 1994). The related folds of HSP20s and p23, together with the experimentally verified chaperoning activities of both, could indicate a similar biochemical function as chaperones and co-regulators of HSP90 activity (Bose *et al.*, 1996). However, we were not able to detect an interaction between RSI2 and the I-2-interacting HSP90 isoform (de la Fuente van Bentem *et al.*, 2005) using yeast two-hybrid assays (results not shown). Likewise, we were unable to establish the presence of a ternary complex of I-2, RSI2 and HSP90 in RSI2 pull-down assays (Figure 2) after probing the blot with an HSP90 antibody (data not shown). Future studies are required to determine whether RSI2 can function as a p23-like regulator of the HSP90/SGT1 machinery or whether it performs its chaperone functions independently of HSP90.

We currently cannot exclude the possibility that the interaction between I-2 and RSI2 is indirect and requires a bridging protein that is functionally conserved in yeast. Future experiments aimed to analyse the RSI2 and I-2 interaction *in planta* and to identify other proteins in this I-2/RSI2 complex using mass spectrometry might provide a partial answer this question. Co-expressing RSI2 and I-2 in *N. benthamiana* via agroinfiltration followed by co-immunoprecipitation could show co-existence of both partners in one complex, but this would not exclude the requirement for a bridging protein. The requirement for a bridging protein could be determined by analysing the dynamic composition of the I-2 protein complex immunoprecipitated from tomato under various infection states, but this strategy requires the development of I-2 antibodies suitable for immunoprecipitation or a protein tag that does not interfere with I-2 function. However, we consider a direct interaction more likely, as suggested in the current model for class I HSP20 function, which proposes direct interaction with client proteins through both the non-conserved N-terminal domain and the conserved ACD (Basha *et al.*, 2006). The strong conservation in the ACD suggests that this region is essential for chaperone activity, whereas the variable N-terminus may be involved in client protein recognition specificity. The RSI2-interacting region was mapped to LRR15–19 of I-2 (Figure 1). This region differs from that required for interaction with HSP90 (LRR1–11), but overlaps with the PP5-interacting region (LRR12–22) (de la Fuente van Bentem *et al.*, 2005), suggesting that these proteins might compete for binding. LRR1–29 and full-length I-2 contain the RSI2 interaction surface, but no interaction was observed in the yeast two-hybrid assay. Similarly, the PP5/I-2 interaction in yeast was observed with truncated versions of I-2 but not with the full-length protein (de la Fuente van Bentem *et al.*, 2005). Possibly, folding of extended LRR domains in yeast differs from that in the plant background, disrupting the interaction surface.

We did not observe an interaction between RSI2 and Mi-1 in pull-down assays (Figure 1), and accumulation of Mi-1 expression *in planta* was not affected by *RSI2* silencing (Figure 3), implying that Mi-1 is not itself a RSI2 client protein. However, Mi-1-mediated HR was compromised by *RSI2* silencing (Figure 2). Possible explanations are that Mi-1 interacts with an RSI2 homologue that is silenced in these plants, or that RSI2 is required for the function of a protein downstream of Mi-1. A possible downstream candidate is the NB-ARC-LRR protein NRC1, which is involved in signalling mediated by many resistance proteins including Mi-1 (Gabriëls *et al.*, 2007). Further studies are required to determine whether NRC1 is indeed an RSI2 client protein.

This report links an HSP20 to accumulation and function of R proteins. Another HSP20 (*Nt*-sHSP) has previously been linked to disease resistance in plants (Maimbo *et al.*, 2007). In *NtsHSP* silenced plants, disease symptoms triggered by *Ralstonia solanacearum* are enhanced and expression of defence-related marker genes is compromised. It remains to be investigated, however, whether this HSP20 is also involved in R protein-mediated resistance, as the HR triggered by a pathogen or the elicitor INF1 was unaffected in these silenced lines (Maimbo *et al.*, 2007). The phylogenetic tree in Figure S1 shows that *Nt-*sHSP is distantly related to RSI2, and further shows that plants possess a large number of highly diverse HSP20s with various subcellular localizations. RSI2 is member of a relatively large cluster of paralogues specific to species of the Solanaceae family (tomato, potato, *Capsicum* and *Nicotiana benthamiana*). The closest non-*Solanaceae* family member in this branch (from *Arabidopsis thaliana*) is relatively distant. A similar clustering of orthologues is seen in other branches of class I, implying that there is strong selection pressure for rapid diversification of the class I HSP20s. It is tempting to speculate that this reflects co-evolution of class I HSP20s with their (rapidly evolving) client proteins. Prime candidates for such client proteins are the R proteins, as these are rapidly evolving and the spectrum of NB-LRR subfamilies differs greatly between plant families (McHale *et al.*, 2006). Co-evolution between specific HSP20s and R proteins is supported by the observed specificity of I-2 for RSI2 reported here. First, we did not retrieve other HSP20 orthologues when we screened a tomato cDNA library (de la Fuente van Bentem *et al.*, 2005). Second, we found that closely related tomato HSP20 homologues did not interact with I-2 in the yeast two-hybrid assay (Figure S2).

A specific role for RSI2-like chaperones in maintaining R protein stability implies that R proteins are generally unstable and prone to inactivation. Support for this idea is the loss of function observed for many R proteins, including Mi-1 (Dropkin, 1969), at elevated temperatures.

Silencing of *HSP90* or *SGT1* also suppresses the ability of auto-active mutants of Mi-1 and I-2 to trigger an HR. The interaction between SGT1 and HSP90 is necessary for SGT1 to fulfil its function in resistance mediated by the R protein Rx (Boter *et al.*, 2007). SGT1 is linked to protein degradation by the ubiquitin/26S proteasome pathway, as it is important for the function of several SCF (for SKP1/CULLIN1/F-box protein) complexes (Kitagawa *et al.*, 1999; Azevedo *et al.*, 2002; Gray *et al.*, 2003). Silencing of the genes encoding SGT1 or its interaction partner HSP90 affected accumulation of full-length I-2 protein, possibly by reduced chaperoning activity. At the same time, the cell may not be able to completely degrade the (partially) unfolded I-2 products because a link to the 26S proteasome is broken by *SGT1* silencing. This may explain the accumulation of intermediate degradation products upon *HSP90* or *SGT1* silencing (Figure 3). Such accumulation was not observed upon *RSI2* silencing, which indicates that RSI2 is involved in stabilizing the I-2 protein but not in its elimination by the 26S proteasome.

To summarize, we report an HSP20 that is a part of the I-2 protein complex *in vitro*, and is required for the HR mediated by auto-active mutants of I-2 and Mi-1 *in planta*. We also demonstrate that RSI2, SGT1 and HSP90 are required for I-2 accumulation. We can thus add RSI2 to the list of chaperones that not only interact with NB-ARC-LRR R proteins but are also required for their function.

## Experimental procedures

### Yeast two-hybrid assays and protein isolation from yeast

Construction of a *Fusarium*-infected tomato cDNA interaction library and the yeast two-hybrid screening method, including the I-2 baits used in this study, have been described previously (de la Fuente van Bentem *et al.*, 2005). The host strain PJ69-4a was transformed with bait (pAS2-1) and prey (pACT2) constructs (Clontech, http://www.clontech.com/) and the presence of both vectors was selected for on minimal medium (MM) plates lacking tryptophan and leucine (–WL). Droplets of a cell dilution corresponding to 10^4^ colony-forming units were spotted on minimal medium plates that also lacked adenine (–AWL) to analyse transcriptional activation of the marker genes that indicate interaction between the bait and prey proteins. To test interaction of the HSP20 orthologues, dilutions corresponding to 10^7^, 10^6^ and 10^5^ colony-forming units were used.

To confirm expression of the HSP20 prey proteins, transformed yeast was grown for 5 days in selective minimal medium (–WL). This culture was used to inoculate 25 ml of YPAD medium (20 g/l Peptone, 20 g/l Dextrose, 10 g/l Yeast extract, 60 mg/l Adenine hemisulphate), and grown for an additional 24 h. Cells were collected and proteins were extracted as described in the Clontech yeast protocol book (http://www.clontech.com/images/pt/PT3024-1.pdf) by adding 100 μl of cracking buffer [8 m urea, 5% w/v SDS, 40 mm Tris/HCl pH 6.8, 0.1 mm EDTA, 0.4 mg/ml bromophenol blue, 2 mm PMSF and 1× protease inhibitor cocktail (Roche, http://www.roche.com)] per 7.5 OD units (600 nm) of yeast cells. Collected cells were broken using glass beads in a Fastprep (2 × 45 sec) (Q-Bio Gene, http://www.qbiogene.com). Ten microlitre aliquots of the resulting protein fractions (supernatant) were loaded on SDS–PAGE gels for immunoblotting. The blots were probed using horseradish peroxidase-conjugated anti-HA antibody (monoclonal 12CA5; Roche) at a 1:1000 dilution, and luminescence was used to visualize the HA-tagged proteins.

### Phylogenetic tree and sequence alignments

SGN sequence data were downloaded from the Unigene (contigs) family 109 HSP20 with 118 family members (http://www.sgn.cornell.edu). SGN contigs were checked for sequencing errors and corrected when possible. The variable N/C-terminal domains flanking the ACD were trimmed even though they contain areas of conservation in sub-clades. Contigs that did not cover the entire HSP20 core were excluded from further analysis. The Arabidopsis and rice sequences were obtained based on cut-off assignment of the presence of the HSP20 protein domain (Pfam HMM model). The evolutionary history was inferred using the minimum evolution method. The distances were computed using the Poisson correction method, and are given as the number of amino acid substitutions per site. The minimum evolution tree was searched using the close neighbour interchange (CNI) algorithm at a search level of 1. Positions containing alignment gaps and missing data were eliminated in pairwise sequence comparisons. There were a total of 194 positions in the final dataset. Phylogenetic analyses were performed using MEGA4 (Tamura *et al.*, 2007). Clade annotations were as described previously (Scharf *et al.*, 2001).

### Vector construction

The binary vectors carrying wild-type I-2, I-2^D495V^, wild-type Mi-1 and Mi-1^D841V^ have been described previously (van Ooijen *et al.*, 2008b). For construction of the TAP-tagged Mi-1 construct, the coding sequence was amplified by PCR on pSE23 (Gabriëls *et al.*, 2007) using primers 5′-AAAAAGCAGGCTCTATGGAAAAACGAAAAGATATT-3′ and 5′-AGAAAGCTGGGTTCTTAAATAAGGGGATATTCTTCTG-3′. Gateway *att*B sequences were added by adapter PCR using the primers 5′-GGGGACAAGTTTGTACAAAAAAGCAGGCT-3′ and 5′-GGGGACCACTTTGTACAAGAAAGCTGGGT-3′. The resulting PCR products were transferred directly to binary vector CTAPi (Rohila *et al.*, 2004) using the Gateway one-tube protocol (Invitrogen, http://www.invitrogen.com/). To create a TAP-tagged auto-active Mi-1 mutant, the *Bsp*119I/*Bcu*I fragment of a tagged wild-type clone was ligated in the corresponding sites of the non-tagged Mi-1 H840A construct, thereby removing the stop codon and allowing a translational fusion to the tag (van Ooijen *et al.*, 2008a). A TAP–GUS fusion control was generated by a Gateway LR reaction using the GUS control plasmid included in the LR kit (Invitrogen) and the binary vector NTAPi (Rohila *et al.*, 2004).

*RSI2* silencing was performed using the tobacco rattle virus (TRV) system by cloning an *Eco*RI/*Xho*I fragment from the cDNA pACT2 library clone (de la Fuente van Bentem *et al.*, 2005) into pYL156 (Liu *et al.*, 2002). An *Eco*RI/*Sal*I fragment from the pAS2-1 bait vector carrying the tomato PP5 tetratricopeptide-repeat (TPR) domain (de la Fuente van Bentem *et al.*, 2005) was ligated into pYL156 digested with *Eco*RI/*Xho*I. A *Bam*HI/*Sal*I fragment from the *SGT1* silencing vector used by Peart *et al.* (2002) was ligated into pYL156 digested with *Bam*HI/*Xho*I. *RAR1* was silenced using the silencing vector described by Liu *et al.* (2002). *HSP90* silencing was performed using the TRV silencing vector described by Ratcliff *et al.* (2001) and the RNA2 clone described by (de la Fuente van Bentem *et al.* (2005).

pGEX-RSI2 was generated by cloning the full-length *RSI2* sequence from the pACTII interaction clone (de la Fuente van Bentem *et al.*, 2005) into pGEX-KG (Guan and Dixon, 1991) digested with *Nco*I/*Xho*I. All clones were validated by sequencing.

Full-length cDNAs of three selected class I HSP20s were PCR-amplified from EST clones (National BioResource Project of Japan) using primers containing either an *Eco*RI or *Xho*I site (underlined) for directional cloning into pACT2. EST clone FC02DH06 (SGN-U312450) was amplified using primers 5′-GGAATTCAAATGTCACTGATCCCAAGAATC-3′ and 5′-GGCTCGAGTTAACCAGAGATCTCAATGGA-3′, clone FB14BB10 (SGN-U312454) was amplified using primers 5′-GGAATTCAAATGTCTCTGATCCCAAGAATT-3′ and 5′-GGCTCGAGTTAACCAGAAATCTCAATGGA, and clone FC08BB05 (SGN-U316206) was amplified using primers 5′-GGAATTCAAATGTCTCTGATTCCAAGCTTC-3′ and 5′-GGCTCGAGTTAACCAGAGATGTCAATTGCC-3′. These clones have been described previously (Tsugane *et al.*, 2005). All clones were sequenced to verify the presence of the correct insertion sequence.

### GST–RSI2 production and pull-down

*Escherichia coli* strain BL21 (DE3) was transformed with empty pGEX-KG vector and with pGEX-KG containing full-length *RSI2*. Protein expression was induced by incubation at room temperature for 5 h in the presence of 1.5 mm IPTG. Pelleted cells (13 000 ***g***) were frozen, and then thawed by resuspending in 10 ml ice-cold PBS, pH 7.4, supplemented with 1× protein inhibitor cocktail (Roche). Lysozyme (1 mg/ml) was added, and the suspension was rotated at 4°C for 30 min, followed by addition of 0.5% v/v Triton X-100 and incubation for a further 30 min. The cell mixture was sonicated, and cell debris was removed by centrifugation at 18 000 ***g*** at 4°C. To 1 ml of crude extract of induced *E. coli* cells, 200 μl of 50% pre-equilibrated glutathione bead slurry (GE Healthcare, http://www.gehealthcare.com) was added, and capture of GST-tagged proteins was performed with rotation at 4°C for 1.5 h. Pelleted beads (100 g) were rinsed four times with 0.5 ml ice-cold PBS, pH 7.4, supplemented with Complete protease inhibitors (Roche). Total protein was extracted from *N. benthamiana* tissue in which either I-2 or TAP-tagged Mi-1 proteins were expressed by *Agrobacterium* transformation. Total plant protein (10 mg) was supplemented with 0.1% v/v NP-40 to obtain an interaction buffer, and added to immobilized GST–RSI2 or GST protein alone. Equal loading of GST–RSI2 and GST (10 μg per pull-down) was confirmed using Bradford protein quantification. The final interaction buffer composition was 25 mm Tris pH 8, 1 mm EDTA, 150 mm NaCl, 5 mm DTT, 0.2% v/v NP-40, 1 x Complete protease inhibitor cocktail (Roche) in a total volume of 1.5 ml. This mixture was incubated with rotation overnight at 4°C to allow binding. The beads were pelleted (100 g) and rinsed five times with ice-cold interaction buffer. Proteins were eluted by addition of Laemmli sample buffer to the pelleted beads and boiling for 5 min. Blotting and immunodetection were performed as described previously (van Ooijen *et al.*, 2008b).

### *Agrobacterium*-mediated transformation and VIGS

*Agrobacterium tumefaciens* strain GV3101(pMP90) (Koncz and Schell, 1986) was transformed with the indicated vectors and infiltrated as described previously (van Ooijen *et al.*, 2008b). Agroinfiltrations were performed in the laboratory (no direct sunlight, 20°C). For infiltration of *Agrobacterium* carrying TRV-based silencing constructs (Ratcliff *et al.*, 2001; Liu *et al.*, 2002), 2-week-old *N. benthamiana* leaves were used. Silencing was performed using the indicated RNA1 and RNA2 constructs, infiltrated at an OD_600_ of 1. Empty RNA2 vectors and their corresponding RNA1 constructs were used as negative controls. Plants were kept in the laboratory for 3 days and then transferred back to the greenhouse.

*Agrobacterium*-mediated expression of the R proteins was performed using a bacterial suspension with an OD_600_ of 1 in the laboratory using 3-week-old *N. benthamiana* plants or 2.5 weeks after silencing. HR was quantified on a scale from 0 to 4 as shown in Figure S6, and cell death was visualized using trypan blue staining as described previously (van Ooijen *et al.*, 2008b).

For analysis of protein expression levels in silenced plants, three infiltrated leaves from three independent silenced plants (total nine) were harvested and pooled 24 h after agroinfiltration. Protein extraction was performed as described previously (van Ooijen *et al.*, 2008b).

### RT-PCR

Total RNA was extracted using Trizol (Invitrogen). cDNA was amplified from 1 μg RNA using SuperscriptI II (Invitrogen) as described by the manufacturer. RSI2 fragments were amplified using the primers 5′-CTGAAGCACATGTGTTTAAGGCC-3′ and 5′-CTTGACATCAGGCTTCTTCAC-3′. The resolving agarose gel was stained using SYBR Green (Invitrogen) and scanned using a STORM phosphoimager (Amersham Bioscience, http://www5.amershambiosciences.com/). The signal intensities were measured using ImageQuant (GE Healthcare) and corrected for background signals.

### One-way anova

For statistical analysis of HR induction by auto-active R protein mutants on silenced plants, HR was visually scored 3 (I-2) or 4 (Mi-1) days after agroinfiltration as described above. Datasets from three independent silencing experiments were scored, totalling the number of replicates indicated in Figure 2. A one-way anova was performed on these data using a significance interval of 95%. Error bars represent a 95% confidence level calculated using Excel.

## Notes

The Genbank accession number for the *RSI2* sequence is AY150040.

## SUPPORTING INFORMATION

**Figure S1.** Evolutionary relationships of 113 HSP20 taxa.

**Figure S2.** Yeast two-hybrid interactions between I-2 and various class IA HSP20 proteins.

**Figure S3.** Coomassie-stained SDS–PAGE gel showing purified GST–RSI2 and GST proteins produced in *Escherichia coli*.

**Figure S4.** C-terminally TAP-tagged Mi-1^H840^ induces the HR.

**Figure S5.** *N. benthamiana* plants silenced for phytoene desaturase.

**Figure S6.** HR intensity scores.

**Appendix S1.** Clustal W (1.8) multiple sequence alignment of the core HSP20 domain used for the phylogeny analysis.

Please note: As a service to our authors and readers, this journal provides supporting information supplied by the authors. Such materials are peer-reviewed and may be re-organized for online delivery, but are not copy-edited or typeset. Technical support issues arising from supporting information (other than missing files) should be addressed to the authors.
