# Supplementary figures and images for: The small heat shock protein 20 RSI2 interacts with and is required for stability and function of tomato resistance protein I-2
Source: Plant J. 2010 Jun 16;63(4):563–72. doi: 10.1111/j.1365-313X.2010.04260.x (PMC2988412; doi:10.1111/j.1365-313X.2010.04260.x)

**A**

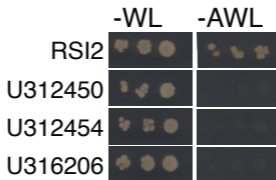

**B**

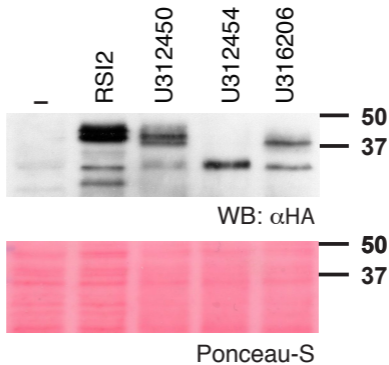

Supplement: Supplementary file 2 [file tpj0063-0563-SD2.pdf]

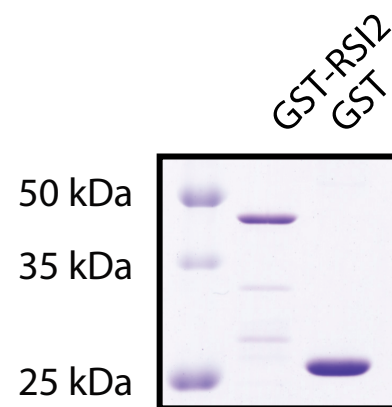

Supplement: Supplementary file 3 [file tpj0063-0563-SD3.pdf]

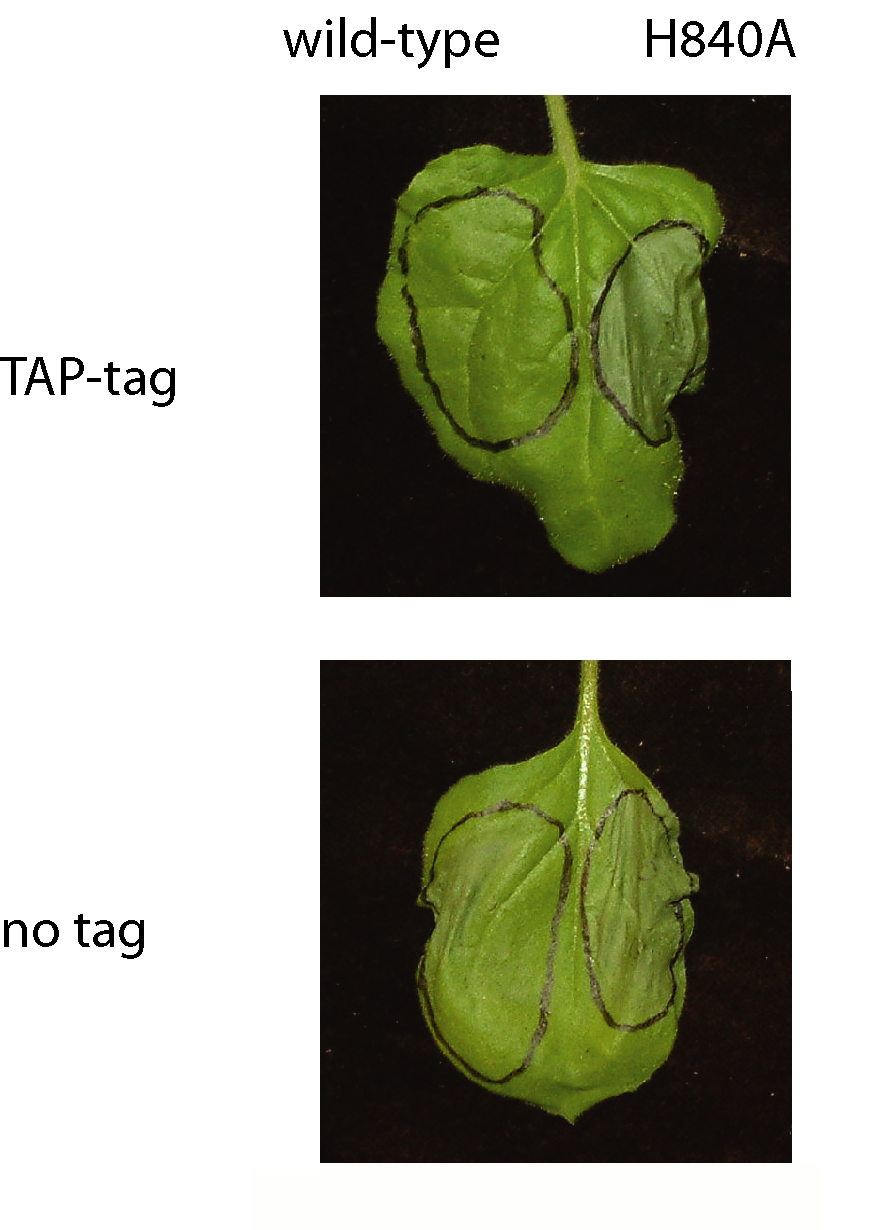

Supplement: Supplementary file 4 [file tpj0063-0563-SD4.tiff]

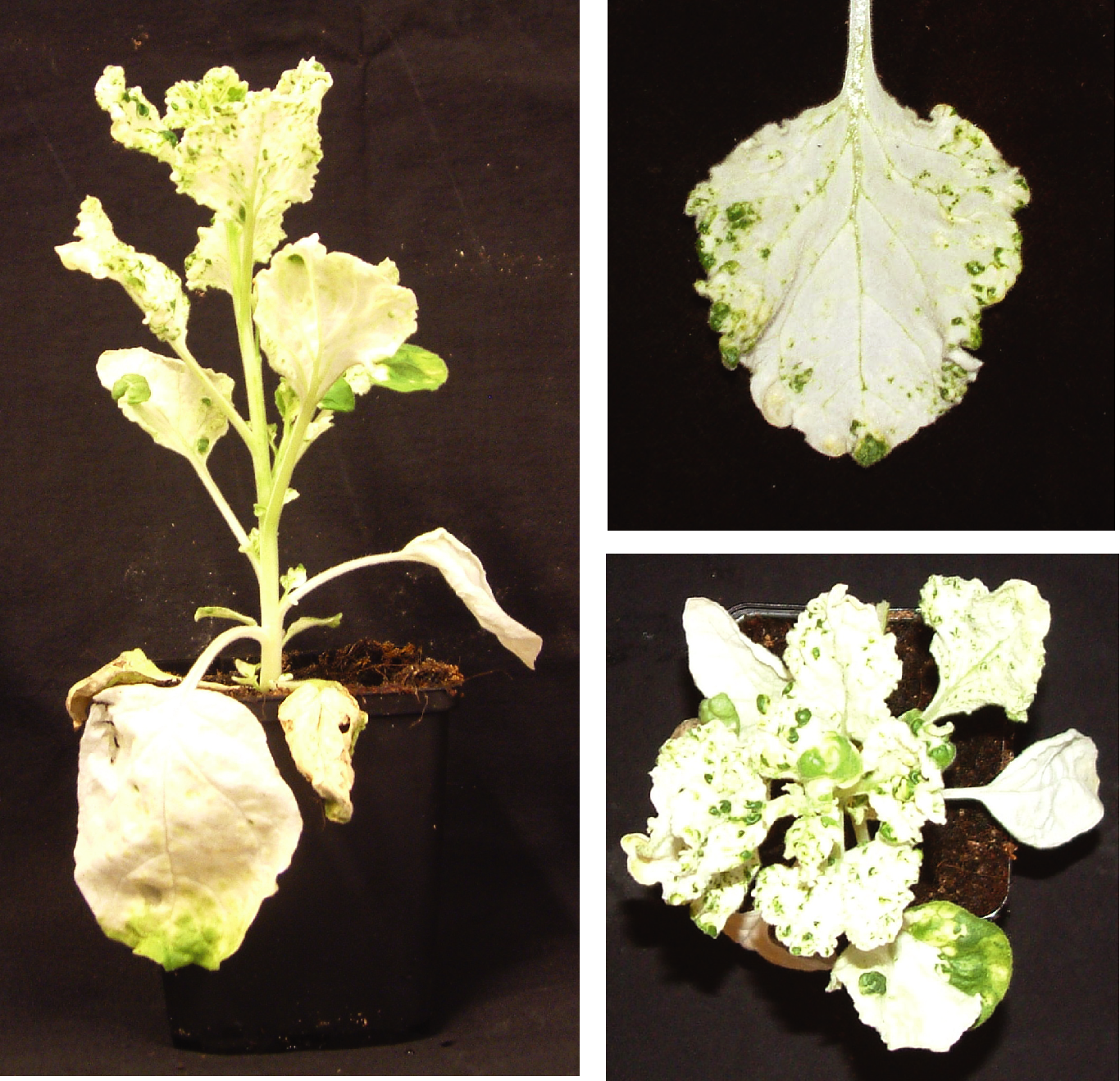

Supplement: Supplementary file 5 [file tpj0063-0563-SD5.tiff]

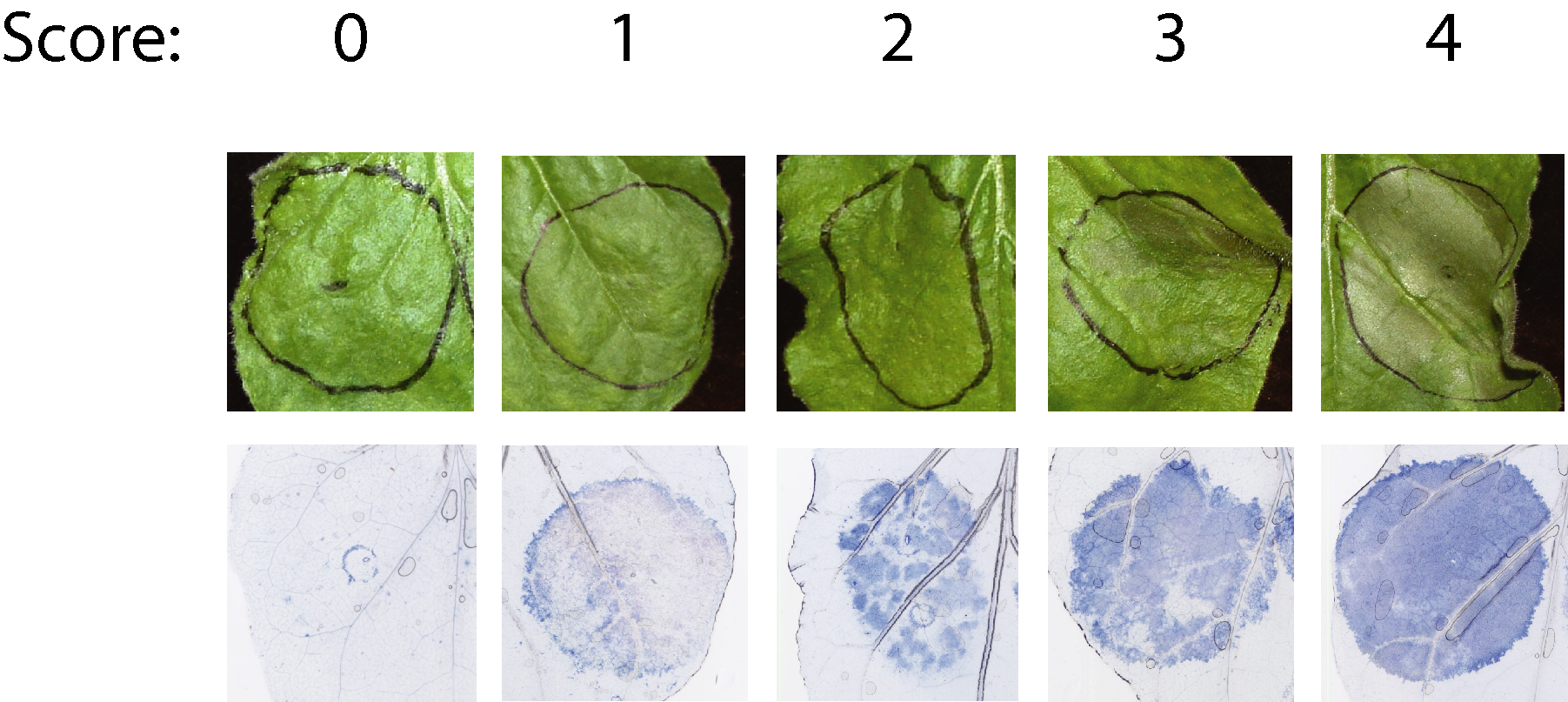

Supplement: Supplementary file 6 [file tpj0063-0563-SD6.tiff]
