# Supplementary material for: The small heat shock protein 20 RSI2 interacts with and is required for stability and function of tomato resistance protein I-2
Source: Plant J. 2010 Jun 16;63(4):563–72. doi: 10.1111/j.1365-313X.2010.04260.x (PMC2988412; doi:10.1111/j.1365-313X.2010.04260.x)
Supplement: Supplementary file 7 [file tpj0063-0563-SD7.doc]

CLUSTAL W (1.8) multiple sequence alignment (BioEdit-generated mock-up)

Sl-SGN-U326583 --------------------SPLLFPYQFIP-ENYVHWRETPESHIYSAD

St-SGN-U274954 --------------------SPLLFPYQFIP-ENYVHWRETPESHIYSAD

At4G21870 --------------------VLASQSLNNYQ-ENHVRWSQSPDSHTFSVD

Sl-SGN-U329160 --------------------GPAARAVYEDF-RPVHERHQDEEAEKLVIY

St-SGN-U293629 --------------------GPAARPVYEDF-RPVYERHQDEEAEKLIIY

Sl-SGN-U323555 --------------------GAAPNQVYEDF-VPTTELVQEQDSDTLLLD

At5G20970 --------------------NFGIERVYQEF-EPATRWTSEPDAEVLVAD

At2G27140 --------------------HANANRIYDEF-EPLSNWKTEQGFEALTIY

At5G04890 --------------------GTGFGVQYEDF-VPKSEWKDQPEATILNID

Sl-SGN-U340504 --------------------TTSVNPVYEDI-EPSSGWIEDAENHYLLID

Sl-SGN-U312454 --------------------GTNSGESSAFA-NTRIDWKETPEAHVFKAD

St-SGN-U269409 --------------------GTNSGETSAFA-NTRIDWKETPEAHVFKAD

Sl-SGN-U312453 --------------------STNSGESSAFA-NTRIDWKETPEAHVFKVD

Nb-DQ275464 -------------------------ETSAFA-NTRVDWKETPEAHVFKAD

Sl-SGN-U333698 --------------------STNSGESSAFA-NTRIDWKETPEAHVFKVD

Sl-SGN-U312455 --------------------GTNSGETSAFA-NTRIDWKETPEAHVFKAD

St-SGN-U269408 --------------------FSNSGETSAFA-NTRIDWKETPEAHVFKAD

Ca-SGN-U196343 --------------------GFNSGETSTFA-NARVDWKETPEAHVFKAD

Ca-SGN-U196341 --------------------GSNSREASAFA-NTRIDWKETPEAHVFKAD

St-SGN-U269410 --------------------VSNSGETSAFA-NTRIDWKETPEAHVFKAD

Sl-SGN-U312450 --------------------VSNSGETSAFA-NTRIDWKETPEAHVFKAD

Ca-SGN-U196342 --------------------GSSSRETSAFA-NTRIDWRETPEAHVFKAD

Sl-SGN-U333697 --------------------GTNSGESSAFA-NTRIDWKETPEAHVFKAD

St-SGN-U287373 -------------------QFPIQGETSAFA-NTRVDWKETPEAHVFKAD

At1G59860 --------------------SSSS---SAIA-NARVDWKETAEAHVFKAD

At1G07400 --------------------SSLSGETSAIT-NARVDWKETAEAHVFKAD

At2G29500 --------------------SSLSRENSAIV-NARVDWRETPEAHVFKAD

At3G46230 -------------------NAP-AKDVAAFT-NAKVDWRETPEAHVFKAD

At1G53540 -------------------NAP-AMDVAAFT-NAKVDWRETPEAHVFKAD

At5G59720 -------------------NASTARDVAAFT-NARVDWKETPEAHVFKAD

St-SGN-U269225 -------------------PPSSVHETSVFA-NAKIDWKETPEAHVFKVD

St-SGN-U269224 -------------------PPSSVRETSVFA-NARIDWKETPEAHVFKVD

Sl-SGN-U316206 --------------------PSSAREISAFA-NAKIDWKETPQAHVFKVD

Sl-SGN-U316208 --------------------PYSAPEISDFA-NAKIDWKETPESHVFKVD

Sl-SGN-U316205 --------------------PSSAREISAFA-NAKIDWKETPQAHVFKVD

Sl-SGN-U316207 --------------------PSSAREISAFA-NAKIDWKETPQAHVFKVD

St-SGN-U269763 --------------------PSSTREISAFA-NAKIDWKETPEAHIFKVD

Sl-SGN-U342568 -------------------VPDTARDTSAFA-TARIDWKETPEAHVFKAD

St-SGN-U269765 -------------------VPSSAREVSAFV-NAKMDWKETSEAHIFKVD

St-SGN-U269764 --------------------PSSAREISAFA-NAKIDWKETPEAHVFKVD

St-SGN-U269761 --------------------PSSAREISAFA-NAKIDWKETPEAHVFKVD

St-SGN-U269762 --------------------PSSAREISAFA-NAKIDWKETPEAHVFKVD

Os03g16020 -------------------PRANS-DAAAFA-GARIDWKETPEAHVFKAD

Os02g52150 --------------------PAG-RAGAATL-RRGWNAKESEEALHLRVD

Os06g11610 ------------------AAPGTNGLATAAA-RRGGWWVAKEDDDAVHLK

Os01g04370 --------------------ATSDNDTAAFA-NARIDWKETPESHVFKAD

Os01g04380 --------------------ATSDNDTAAFA-NARIDWKETPESHVFKAD

Os03g16030 -------------------PRGTSSETAAFA-GARIDWKETPEAHVFKAD

Os03g16040 -------------------PRANS-DAAAFA-GARIDWKETPEVHVFKAD

Os03g15960 -------------------PRGASSETAAFA-GARIDWKETPEAHVFKAD

Os01g04340 -------------------VRSLAERCPVLT-NVRVDWKETPTAHVFTAD

Os01g04350 -------------------ADAWLASDTSAFANTYIESRETAEAYVFRAD

Os02g03570 -------------------PSAAAAAAGVPS-TASMDWKETAAAHVFMAD

Os04g36750 ----------------------DRDDVAMLS-MARVDWRETGDAHEVVVD

Os11g13980 ------------------------GEPAAVA-LARCDWKETPEAHVVTVD

Os06g14240 ---------------------FPPDWASASA-TAAMDWVETPTSHVLRIN

Os01g08860 -------------------TRAYVRDARAMA-ATPADVKDLPGAYAFVVD

Os02g54140 --------------------HGQHRISGIGG-GAPVDIMETPGEYAFVLD

Os02g12610 ------------------TRAYVRDRRAMANTPMDVKELRASGALVLAVD

Sl-SGN-U333694 ------------------FPGSNSGGRFAFA-HPRIGWKGTPEGHVFKGD

Sl-SGN-U333693 ------------------FPSPIQGKLCIA--NPPNRLKETPEAQVFQVD

Ca-SGN-U197520 -------------------PPPLLVKPLLS--KCKNRLERDPTSAHLQSR

St-SGN-U273600 -------------------PIVGMMNMCPVL-STPIDWKETPQAHCFFVD

St-SGN-U288775 -------------------PIVGMMNMCPVL-STPIDWKETPQAHCFFVD

Sl-SGN-U321313 -------------------LSPTIHRSFSGS-PALLDWIESPNSHIFKIN

St-SGN-U298363 ---------------------------------XLLDWIESPNAHIFKIN

At5G37670 -------------------PFRRFQEWSRS--TALIDWMESNNSHIFKIN

Sl-SGN-U325739 -------------------GFRNDEISALA--HASVDWRETDQAHVFLVD

Sl-SGN-U332227 -------------------VE-SIA-------LTRSDWKETATEHVITLD

Sl-SGN-U341636 -------------------IDQTIAL------LARSDWKETSKEHIISLD

Sl-SGN-U318540 -------------------LENTL--------LARVDWKETAKGHVISVE

St-SGN-U281516 -------------------LENREETTLPLS-IARVDWKETAEGHVISID

At4G10250 -------------------LERDTSVALS---PARVDWKETAEGHEIMLD

Sl-SGN-U332716 -------------------LVSPSRSNESRG-TIPVDILDTPKEYIFYMD

At1G54050 -------------------SNNESRGRGSSN-NIPIDILESPKEYIFYLD

At5G12020 -------------------SRVYMRDAKAMA-ATPADVIEHPNAYAFVVD

At5G12030 -------------------SRAYMRDAKAMA-ATPADVIEHPDAYVFAVD

Sl-SGN-U316987 -------------------SRNYVRDAKAMA-ATPADVKEYPNSYVFVVD

St-SGN-U271074 -------------------SRNYVRDAKAMA-ATPADVKEYPNSYVFVVD

Sl-SGN-U316986 -------------------SKKYVRDAKAMA-ATPVDVKEYPDSYVFVVD

Sl-SGN-U335906 -------------------SKKYVRDAKAMA-ATPVDVKEYPDSYVFVVD

Ca-SGN-U196893 -------------------SRNYVRDAKAMA-ATPVDVKEYPNSYVFVVD

St-SGN-U271057 -------------------APAPSFHHVPAT-MAQIEYKETPEAHIFRCN

Sl-SGN-U321049 -------------------APAPSFHHVPAT-MAQIEYKETPESHIFRCN

Ca-SGN-U207893 -------------------APPPTFHHGPSS-MVQIESKDTDEAHIFRAN

Sl-SGN-U321004 -------------------APSLSFPHVTPF-LAQFECNVTPEAYVFRAN

St-SGN-U298302 -------------------APSLSFPREPQS-IAQIEYKATQEAHIFKAN

St-SGN-U273896 -------------------NKIFDNLNLNPS-QLLGRYKEDDKNYKIRYD

Sl-SGN-U321169 -------------------NKIFDNFNLNPS-QLLGRYKEDDKNYKIRYD

At1G52560 -------------------NRIFDNFNVNPF-QLMGQVKEQDDCYKLRYE

Sl-SGN-U314000 -------------------GRNR-ASGTGEI-RTPWDIHDDENEIKMRFD

Sl-SGN-U334715 -------------------GRNR-ASGTGEI-RTPWDIHDDENEIKMRFD

St-SGN-U274067 -------------------GRNR-ASGAGEI-RTPWDIHDDENEIKMRFD

At4G27670 -------------------GRNRGGSGVSEI-RAPWDIKEEEHEIKMRFD

Sl-SGN-U339823 -------------------GPSTDDIGYRRG-RTPWEIKENEGEYKMRFD

St-SGN-U276891 -------------------GPSTDDVGYKRG-RTPWEIKESEAEYKMRFD

Sl-SGN-U339822 -------------------GASTDDIGYRRG-RTPWEIKENEGEYKMRFD

St-SGN-U271467 -------------------AAPRAMGAGVGA-RRGWDVKEDDNALYIKMD

St-SGN-U288169 -------------------AAPRAMGAGVGA-RRGWDVKEDDNALYIKMD

Sl-SGN-U312456 -------------------AAPRAMGAGVGA-RRGWDVKEDDNALYIKMD

Ca-SGN-U196718 -------------------AAPRSMGAVVPS-RRGWDVREDADALYIKMD

At4G25200 -------------------SATRGMGA-SGA-RRGWDIKEKDDALYLRID

At5G51440 -------------------SATRGMGA-SGV-RRGWNVKEKDDALHLRID

At2G19310 -------------------REIFPQTSSSTV-NTQLNWTETPTAHVFKAY

At2G03020 -------------------LQPHPLAFPQPT-MAYESKQLPNGKLFVRAD

At4G16540 ----------------------SLCFCQQFL-VAQVSVKLPNGKLFVRAD

Nt-shsp(Ralstonia) -------------------GTAEKCAIGPS--LGVVDIGNSENAYLFRVA

Nb-shsp(Ralstonia) -------------------GTAEKCAIGPS--LGVVDIGNSENAYLFRVA

At5G47600 -------------------SPIRNRFQKSGS-QAVYEVTETKKSCVTRVD

At1G06460 -------------------DLPKLANLGTVW-SPRSNVAESTHSYVVAIE

At1G12180 -------------------NYKLEIKEYGGG-KGPVRWFFKEDSCVAMID

Sl-SGN-U329774 -------------------FKMENPFQSAGP-KEVLEVDTLKDGILVRVA

At5G47590 -------------------EDHPCLVSGRKG-ALIG-VTKAGGGAHFAVD

Sl-SGN-U326583 L--PGVKKEEIKVEVEDS-SYLII--RTEAANET----------------

St-SGN-U274954 L--PGVKKEEIKVEVEDS-SYLII--RTEAANET----------------

At4G21870 L--PGLRKEEIKVEIEDS-IYLII--RTEATPMS----------------

Sl-SGN-U329160 L--PGFMKENIRVSTEGK-NTVRV--RGERFVGG---------------N

St-SGN-U293629 L--PGFMKESIRVSSEGK-NTARV--RGERVVGG---------------N

Sl-SGN-U323555 L--TGFRKEQVRVQLTRT-GVVKI--SGQRPVAE---------------N

At5G20970 L--PGFKKEQLKVSVTAT-RKLRL--TGERPTGG---------------N

At2G27140 L--PGFRKEQLKVQVTTT-RKLRV--MGDRPAGA---------------N

At5G04890 L--TGFAKEQMKVTYVHSSKMIRV--TGERPLAN---------------R

Sl-SGN-U340504 L--PGFKREEVKLEVDTF-DNIKV--SGERKVGE---------------N

Sl-SGN-U312454 L--PGLKKEEVKVEVEED-R-LQI--SGERNVEK--EDKND---------

St-SGN-U269409 L--PGLKKEEVKVEVEED-RVLQI--SGERNVEK--EDKND---------

Sl-SGN-U312453 L--PGLKKEEVKVEVEED-RVLQI--SGERNVEK--EDKND---------

Nb-DQ275464 L--PGLKKEEVKVEIEDD-RVLQI--SGERNVEK--EDKND---------

Sl-SGN-U333698 L--PGLKKEEVKVEVEED-RVLQI--SGERNVEK--EDKND---------

Sl-SGN-U312455 L--PGLKKEEVKVEIEED-RVLQI--SGERNVEK--EDKND---------

St-SGN-U269408 L--PGLKKEEVKVEIEED-RVLQI--SGERNVEK--EDKND---------

Ca-SGN-U196343 L--PGLKKEEVKVEIEED-RVLQI--SGERNVEK--EDKND---------

Ca-SGN-U196341 L--PGLKKEEVKVEIEEH-RVLQI--SGERNEEK--EDKSD---------

St-SGN-U269410 L--PGLKKEEVKVEIEED-RVLQI--SGERNVEK--EDKND---------

Sl-SGN-U312450 L--PGLKKEEVKVEIEED-RVLQI--SGERNVEK--EDKND---------

Ca-SGN-U196342 L--PGLKKEEVKVEIEED-RVLQI--SGERNVEK--EDKND---------

Sl-SGN-U333697 L--PGLKKEEVKVEVEED-RVLQI--SGERNVEK--EDKND---------

St-SGN-U287373 L--PGLKKEEVKVEIEED-RVLQI--SGERNVEK--EDKND---------

At1G59860 L--PGMKKEEVKVEIEDD-SVLKI--SGERHVEK--EEKQD---------

At1G07400 L--PGMKKEEVKVEIEDD-SVLKI--SGERHVEK--EEKQD---------

At2G29500 L--PGLKKEEVKVEIEED-SVLKI--SGERHVEK--EDKND---------

At3G46230 V--PGLKKEEVKVEVEDG-NILQI--SGERSSEN--EEKSD---------

At1G53540 L--PGLRKEEVKVEVEDG-NILQI--SGERSNEN--EEKND---------

At5G59720 L--PGLKKEEVKVEVEDK-NVLQI--SGERSKEN--EEKND---------

St-SGN-U269225 V--PGIKKEEVKVEVEEG-RILQI--SGERSKEQ--EEKND---------

St-SGN-U269224 V--PGIKKEEVKVEVEEG-RILQI--SGERSKEQ--EEKND---------

Sl-SGN-U316206 V--PGIKKEEVKVEVEEG-RILQI--SGERSREK--EEKND---------

Sl-SGN-U316208 V--PGIKKEEVKVEVEEG-RILQI--SGERNREK--EEKND---------

Sl-SGN-U316205 V--PGINKEEVKVEVEEG-RILQL--SGERSREK--EEKND---------

Sl-SGN-U316207 V--PGIKKEEVQVEVEEG-RILQI--RGERSREK--KEKND---------

St-SGN-U269763 V--PGIKKEEVKVEVEEG-RILQI--SGERSREQ--EEKND---------

Sl-SGN-U342568 L--PGLKKEEVKVEVEDG-QVLQI--SGERSREQ--EEKND---------

St-SGN-U269765 V--PGIKKEEVKVEVEEE-GILQI--SGEEAENK--RRRMI---------

St-SGN-U269764 V--PGIKKEEVKVEVEEE-GFYKL--AVREAENK--RRRTI---------

St-SGN-U269761 V--PGIKKEEVKVKWKKE-GFYRL--AVREAESK--KRRPI---------

St-SGN-U269762 V--PGIKKEEVKVKWKKE-GFYRL--AVREAESK--KRRTI---------

Os03g16020 V--PGLKKEEVKVEVEDG-NVLQI--SGERIKEQ--EEKTD---------

Os02g52150 M--PGLGKEHVKVWAEQN-SLVIK---GEGEKEA--GEDEG---------

Os06g11610 VSMPGLGKEHVKVWAEQN-SLVIK---GEGEKDP--EDDAD---------

Os01g04370 L--PGVKKEEVKVEVEEG-NVLVI--SGQRSKEK--EDKND---------

Os01g04380 L--PGVKKEEVKVEVEEG-NVLVI--SGQRSKEK--EDKND---------

Os03g16030 V--PGLKKEEVKVEVEDG-NVLQI--SGERSKEQ--EEKTD---------

Os03g16040 V--PGLKKEEVKVEVDDG-NILQI--SGERSREQ--EEKSD---------

Os03g15960 V--PGLKKEEVKVEVDDG-NILQI--SGERNKEQ--EEKTD---------

Os01g04340 L--PGVRKDQAKVEVEDG-GVLVI--SGERAREEDVDGKNDE--------

Os01g04350 L--PAGVKKEEVRVEVDEGNVLVI--TGERSVRR--EEKGQ---------

Os02g03570 M--PGVRREEVRVEVEEE-KVLRI--SGQRARAA--EEKGE---------

Os04g36750 V--PGMRKEDLRVEVEDN-RVLRI--SGERRREETTEQKGG---G----D

Os11g13980 V--PGVRRGDVRVEVDEASRVLRV--SGERRRAGAAEEEEGERDGV----

Os06g14240 V--PGLGKDDVKVQVEDG-NVLTV--RGAAPHAAAEKEREREKDV-----

Os01g08860 M--PGLKSSDIKVQVEEE-RLLVI--SGERRRGGGEEEKEESC-------

Os02g54140 V--PGLSKSDIQVTLEED-RVLVMKSSNGAGNGKRKREEEEGE-C-----

Os02g12610 M--PGVAPADVRVEVEDG-NVLAI--SGERRRPAGDGDDGGEGV------

Sl-SGN-U333694 F--PGVKKGEVKVEVEGD-RVFQI--SGERDVGK--EDKND---------

Sl-SGN-U333693 P--PGLKKEEVQVEVEED-RVPPI--SGERNVEK--EDKDD---------

Ca-SGN-U197520 R--SGDQERGSESEVEEG-RILQI--SGERSREQ--EEKND---------

St-SGN-U273600 L--PGLSKEDVKVEVDNG-RVLKI--SGKWKAEVEELVDENEEKKKKSDV

St-SGN-U288775 L--PGLSKEGVKVEVDNG-RVLKI--SGKWKAEVDENEKSK-----KLDV

Sl-SGN-U321313 V--PGYSKEDIKVQVEDG-NVLVVKAEGHGGKKDEFHGKEK-------DI

St-SGN-U298363 V--PGYSKEDIKVQVEDG-NALVVKAEGHGGKKDEFHGKDK-------DI

At5G37670 V--PGYNKEDIKVQIEEG-NVLSI--RGEGIKEE--KKENL---------

Sl-SGN-U325739 I--PGVKKEDLKVQLEDN--ILEI--SGERVKEE--EKGDD---------

Sl-SGN-U332227 I--PGMKKEDVKIEVEEN-RVLRV--SGERKTEE--EIEGE---------

Sl-SGN-U341636 I--PGMKKEDIKIEVEEN-RVLRI--SGERKTEE--ENIESE--------

Sl-SGN-U318540 V--PGLNKDDIKIEIEEN-RVLRV--SGERKKEE--EKNDEEN-------

St-SGN-U281516 V--PGLKKDDIKIEIEEN-RVLRV--SGERKKEE--EKNDEQN-------

At4G10250 I--PGLKKDEVKIEVEEN-GVLRV--SGERKREE--EKKGD---------

Sl-SGN-U332716 V--PGLSKSDIQVSVEDEKTLVIR-SNGKRKREE-SEEEGC---------

At1G54050 I--PGISKSDIQVTVEEERTLVIK-SNGKRKRDDDESEEGS---------

At5G12020 M--PGIKGDEIKVQVEND-NVLVV--SGERQREN-KENEGV---------

At5G12030 M--PGIKGDEIQVQIENE-NVLVV--SGKRQRDN-KENEGV---------

Sl-SGN-U316987 M--PGLKSGDIKVQVEED-NVLLI--SGERKREE--EKEGA---------

St-SGN-U271074 M--PGLKSGDIKVQVEED-NVLLI--SGERKREE--EKEGA---------

Sl-SGN-U316986 M--PGLKSGDIKVQVEED-NVLLI--SGERKREE--EKEGV---------

Sl-SGN-U335906 M--PGLKSGDIKVQVEED-NVLLI--SGERKREE--EKEGV---------

Ca-SGN-U196893 M--PGLKSGDIKVQVEEE-NVLLI--SGERKREE--EKEGA---------

St-SGN-U271057 L--HGYKKEDVKVQVEDE-KILKI--TGETRMMK---KED----------

Sl-SGN-U321049 L--HGYKKEDVKVQVEDE-KILKI--TGETRMMK---KED----------

Ca-SGN-U207893 L--HGYKKEEVKVQVEDD-RVLKI--TGEKKMRK--EDNHN---------

Sl-SGN-U321004 NL-HGYKKEEVKVQVEDD-RILKI--SGEKKIVE---KEYD---------

St-SGN-U298302 L--HGYKKEEVKVQVENN-KVLKI--SGEKKIVQ---KEYD---------

St-SGN-U273896 V--PGLGKNDVKIMVEDG-ILTIK--GEHKEEKE--EEGSD---------

Sl-SGN-U321169 V--PGLGKKDVKIMVEDG-ILTIK--GEHKQEKE--EEGSD---------

At1G52560 V--PGLTKEDVKITVNDG-ILTIK--GDHKAEEE-KGSPEE---------

Sl-SGN-U314000 M--PGLSKEDVKVSVEND-MLVIK--GEHKKEED--GR------------

Sl-SGN-U334715 M--PGPSKEDVKVSVEND-RPVRK--GEHKKEEG--GR------------

St-SGN-U274067 M--PGLSKEDVKVSVENN-VLVIK--GEHKKEEG--GK------------

At4G27670 M--PGLSKEDVKISVEDN-VLVIK--GEQKKEDS--DD------------

Sl-SGN-U339823 M--PGMTKEDVKVWLEEK-MLVVK--GEKMVKNN--EK------------

St-SGN-U276891 M--PGMTKEDVKVWLEEK-MLVVK--AEKMVKNN--GI------------

Sl-SGN-U339822 M--PGMTKEDVKVWLEEK-MLVVK--GEKMVKNN--EK------------

St-SGN-U271467 M--PGLDKENVKVAVEEN-TLIIK--GEGEKESE--NEE-----------

St-SGN-U288169 M--PGLDKENVKVAVEEN-TLIIK--GEGEKESE--NEE-----------

Sl-SGN-U312456 M--PGLDKENVKVAVEEN-TLIIK--GEGEKESE--NEE-----------

Ca-SGN-U196718 M--PGLDKDNVKVAVEQN-TLIIK--GEGEKESE--DEE-----------

At4G25200 M--PGLSREDVKLALEQD-TLVIR--GEGKNEEDGGEEGESG--------

At5G51440 M--PGLSREDVKLALEQN-TLVIR--GEGETEEG--EDVSG---------

At2G19310 L--PGVDQDEVIAFVDEE-GYLQI--CTGDN-------------------

At2G03020 M--PGVPKENFTVSVTN--GRVKV--TGQAPAVSHD--------------

At4G16540 M--PGVPKENFTVSVTN--GRVKV--TGEAPALSHD--------------

Nt-shsp(Ralstonia) L--PGVRNKCNIKCDIQREGRVRI--EGVVTESDVLKNSSKGYE------

Nb-shsp(Ralstonia) L--PGVRNKCNIKCDIQREGRVRI--EGVITESDVLKNSPKDYE------

At5G47600 M--PGCPESDLTYWVDAN-NVHFF--ADEPAMPEYE--------------

At1G06460 L--PGASINDIRVEVDNT-NLTVT--GRRTSICQKVDAGTK---------

At1G12180 V--PGCSVAPSSYSLEKT-HVGFQ--TEEVDQNNHS--------------

Sl-SGN-U329774 M--PSVGEDGIKVWLENN-TVYFT--GKGDIEVESEE-------------

At5G47590 M--PGVSPDDVEVYADEK-EIRFK--AEIKDVYEH---------------

Sl-SGN-U326583 ----TE-------PIRFMR-KFRLP-GMVD-MDGISASYRD--GVLTVTV

St-SGN-U274954 ----TE-------PIRFMR-KFRLP-GMVD-MDGISASYRD--GVLTVTV

At4G21870 ---PPDQ------PLKFKR-KFRLP-ESID-MIGISAGYED--GVLTVIV

Sl-SGN-U329160 KWHFQE--------------DFQAP-DDCN-MRGIHAKFEN--GILIITM

St-SGN-U293629 TWHFQE--------------DFHAP-DDCN-MRGIHAKFEN--GILIITM

Sl-SGN-U323555 KWLFQK--------------DFPVS-QNCD-RTKISAKFEN--GILYVKQ

At5G20970 KWIFHQ--------------EIPVP-LTVD-IDSVSAMFKD--NKLYIRH

At2G27140 KWIFRK--------------EFPIP-PNID-VDSVSAKFEG--ANLVVRL

At5G04890 KWSFNE--------------VFTVP-QNCL-VDKIHGSFKN--NVLTITM

Sl-SGN-U340504 KFIFQK--------------STIAP-EKSK-SEDTSARIED--GILFVII

Sl-SGN-U312454 KWHRVERS-----SGKFMR-RFRLP-ENAK-MDQVKASMEN--GVLTVTV

St-SGN-U269409 KWHRVERS-----SGKFMR-RFRLP-ENAK-MDQVKASMEN--GVLTVTV

Sl-SGN-U312453 KWHRMERS-----SGKFMR-RFRLP-ENAK-MDQVKASMEN--GVLTVTV

Nb-DQ275464 TWHRVERS-----SGKFMR-RFRLP-ENAK-MDQVKAAMeN--GVLTVTV

Sl-SGN-U333698 KWHRMERS-----SGKFMR-RFRLP-ENAK-MDQVKASMEN--GVLTVTV

Sl-SGN-U312455 TWHRVERS-----SGKFMR-RFRLP-ENAK-MDQVKASMEN--GVLTVTV

St-SGN-U269408 TWHRVERS-----SGKFMR-RFRLP-ENAK-MDQVKASMES--GVLTVTV

Ca-SGN-U196343 TWHRVERS-----SGKFMR-RFRLP-ENAK-MEQVKASMEN--GVLTVTV

Ca-SGN-U196341 TWHRMERS-----SGKFMR-RSRLP-ENAK-MDQVKASMEK--GVLTVTV

St-SGN-U269410 TWHRVERS-----SGKFMR-RFKLP-ENAK-MDQVKASMEN--GVLTVTV

Sl-SGN-U312450 TWHRVERS-----SGKFMR-RFRLP-ENAK-MDQIKASMEN--GVLTVTV

Ca-SGN-U196342 TWHRMERS-----SGKFMT-RFRLP-ENAK-MDQVKASMEN--GVLTVTV

Sl-SGN-U333697 KWHRVERS-----SGKFMR-RFRLP-ENAK-MDQVKASMEN--GVLTVTV

St-SGN-U287373 KWHRVERS-----SGKFMR-RFRLP-ENAK-MDQVKASMEN--GVLTVTV

At1G59860 TWHRVERS-----SGGFSR-KFRLP-ENVK-MDQVKASMEN--GVLTVTV

At1G07400 TWHRVERS-----SGQFSR-KFKLP-ENVK-MDQVKASMEN--GVLTVTV

At2G29500 TWHRVERS-----SGQFTR-RFRLP-ENVK-MDQVKAAMEN--GVLTVTV

At3G46230 TWHRVERS-----SGKFMR-RFRLP-ENAK-VEEVKASMEN--GVLSVTV

At1G53540 KWHRVERS-----SGKFTR-RFRLP-ENAK-MEEIKASMEN--GVLSVTV

At5G59720 KWHRVERA-----SGKFMR-RFRLP-ENAK-MEEVKATMEN--GVLTVVV

St-SGN-U269225 QWHRMERS-----SGKFVR-RFRLP-ENVK-MEEIKAAMEN--GVLTVTV

St-SGN-U269224 QWHCMERS-----SGKFVR-RFKLP-ENVK-MEEIKAAMEN--GVLTVTV

Sl-SGN-U316206 QWHRMERS-----SGKFKR-RFRLP-ENAK-TGEIKAEMEN--GVLTVTV

Sl-SGN-U316208 QWHRMERS-----SGKFIR-RFRLP-ENAK-TGEIKAAMEN--GVLTVTV

Sl-SGN-U316205 KWHRMERS-----SGKFLR-RFRLP-ENAK-TGEIKAAMEN--GVLTVTV

Sl-SGN-U316207 QWHRMERS-----SGKFLR-RFRLP-ENAK-TGEIKAAMEN--GVLTVTV

St-SGN-U269763 QWHRMERS-----SGKFLR-RFRLP-ENAK-TGEIKAAMEN--GVLTVTV

Sl-SGN-U342568 KWHXLGGEE----QRQVPA-QVQAA-GEAK-VDKVNASMEN--GVLTVSV

St-SGN-U269765 SGTVWRGV-----AEVFKE-IQAA--GEYK-DGRNKGSDGE--WGATVTV

St-SGN-U269764 NGTVWRGV-----AEVFKE-IQVA--GECK-DGRNKGSDGE--WGATVTV

St-SGN-U269761 SGPVWRGV-----AEVFKE-IQTA--GKYE-EGEIKAAMEN--GVLTVTV

St-SGN-U269762 SGTVWRGA-----AVKFMR-RFRLP-ENTK-TGEIKAAMEN--GVLTVTV

Os03g16020 KWHRVERS-----SGKFLR-RFRLP-ENTK-PEQIKASMEN--GVLTVTV

Os02g52150 -----AA------PARYSG-RIELAPEVYR-MDQIKAEMKN--GVLKVVV

Os06g11610 -----AA------PPRYTR-RIELPADAFK-MDKIKAEMKN--GVLRVAV

Os01g04370 KWHRVERS-----SGQFMR-RFRLP-ENAK-VDQVKAGLEN--GVLTVTV

Os01g04380 KWHRVERS-----SGQFMR-RFRLP-ENAK-VDQVKAGMEN--GVLTVTV

Os03g16030 KWHRVERS-----SGKFLR-RFRLP-ENTK-PEQIKASMEN--GVLTVTV

Os03g16040 KWHRVERS-----SGKFLR-RFRLP-ENTK-PEQIKASMEN--GVLTVTV

Os03g15960 QWHRVERS-----SGKFLR-RFRLP-DNAK-PEQIKASMEN--GVLTVTV

Os01g04340 RWHHVERS-----SGKFQR-RFRLP-RGAR-VDQVSASMDN--GVLTVTV

Os01g04350 RSHHIERS-----CATFFG-RFHLP-DDAV-VDLVRASMDG--GMLTVTV

Os02g03570 RWHRVERS-----SERFVR-TVRLP-PNAN-TDGVHAALDN--GVLTITI

Os04g36750 HWHREERS-----YGRFWR-QLRLP-DNAD-LDSIAASLDN--GVLTVRF

Os11g13980 RWHRAERA-----AGRFWR-RFRMP-PGAD-VGRVAARLDD--GVLTVTV

Os06g14240 VWHVAERG-----RPEFAR-EVALP-AEVR-VEQIRASVDN--GVLTVVV

Os01g08860 KYLRMERR-----MGKFMR-KFVLP-DNAD-VDKISAVCQD--GVLTVTV

Os02g54140 KYIRLERRA----SPRAFARKFRLP-EDAD-TGGISARCEN--GVLTVTV

Os02g12610 KYLRMERR-----MGKFMR-RFPLP-ESAD-LDGVRAEYKD--GVLTVTV

Sl-SGN-U333694 QWPCVEGS-----SGKFMG-GFRLP-GNAK-MGQVKASMGN--GVLSVSV

Sl-SGN-U333693 KWPCLERS-----RRKFLR-RFRLP-EDAK-MDQGKAFMEN--GMPSGSG

Ca-SGN-U197520 QWHRMERS-----SGKFLR-RFRLP-ENTK-TGEIKAAMED--GVLTVTV

St-SGN-U273600 KWHRVERN-----RGDFCR-KFRLP-QNIK-ADQLKASMEN--GVLIVTV

St-SGN-U288775 KWHRVERN-----RGDFCR-KFRLP-QNIK-AHQLTASMEN--GVLIVTV

Sl-SGN-U321313 VWHVAERG--GGRGGDFSR-EIELP-EDVK-VDQIKAQCEN--GVLTIVV

St-SGN-U298363 VWHVAERG--GGRGGDFSR-EIELP-EDVK-VDQIKAQVEN--GVLTIVV

At5G37670 VWHVAEREAFSGGGSEFLR-RIELP-ENVK-VDQVKAYVEN--GVLTVVV

Sl-SGN-U325739 KWHRVERK-----RGSFCR-KFRLP-ENAN-VEGISCGLEN--GVLTVNV

Sl-SGN-U332227 KWHRAERT-----CGKFWR-QFRLP-GNAD-LEHIKAHLEN--GVLKITV

Sl-SGN-U341636 KWHRVERT-----SGKFWR-QFKLP-RNVD-LEHIKANLDN--GVLKITV

Sl-SGN-U318540 HWHCVERS-----HGKFWR-QFRLP-ENAD-IDTMKAKLEN--GVLTISF

St-SGN-U281516 HWHCVERS-----YGKFWR-QFRLP-ENAD-IDTMKAKLEN--GVLTISF

At4G10250 QWHRVERS-----YGKFWR-QFKLP-DNVD-MESVKAKLEN--GVLTINL

Sl-SGN-U332716 KYVRLERN----PPLKLMR-KFKLP-DYCN-VSAITAKCEN--GVLTVVV

At1G54050 KYIRLERR----LAQNLVK-KFRLP-EDAD-MASVTAKYQE--GVLTVVI

At5G12020 KYVRMERR-----MGKFMR-KFQLP-ENAD-LDKISAVCHD--GVLKVTV

At5G12030 KFVRMERR-----MGKFMR-KFQLP-DNAD-LEKISAACND--GVLKVTI

Sl-SGN-U316987 KFIRMERR-----VGKFMR-KFSLP-ENAN-TDAISAVCQD--GVLTVTV

St-SGN-U271074 KFIRMERR-----VGKFMR-KFSLP-ENAN-TDAISAVCQD--GVLTVTV

Sl-SGN-U316986 KFIRMERR-----VGKFMR-KFSLP-ENAN-TDAISAVCQD--GVLTVTV

Sl-SGN-U335906 KFIRMERR-----VGNFMR-KFSLP-ENAN-TDAISAVCQD--GVLTVTV

Ca-SGN-U196893 KYIRMERR-----VGKFMR-KFTLP-DNAN-TDAISAVCQD--GVLTVTV

St-SGN-U271057 NWHHYERS-----SGKFFT-SFSLP-LNCR-ADYVKSSMEN--GVLTITV

Sl-SGN-U321049 NWHHYERS-----SGKFFT-SFSLP-LNSR-ADYVKSSMEN--GVLTITI

Ca-SGN-U207893 NWHHSEHS-----NGKFFT-SFSLP-KNAK-GDCVKSSMEN--GVLTVTV

Sl-SGN-U321004 NWHHFQKK-----VGKFST-VFNLP-EDAG-VDKVISTMEK--EVLIVTI

St-SGN-U298302 NWHHFQRR-----NGNYFT-AFNLP-EDAK-VDKVKSSMEN--GVLVVTV

St-SGN-U273896 DEFWSSRS-----YGYYNN-SIVLP-EDAK-VDEIKAEMKD--GVLTITI

Sl-SGN-U321169 DEFWSSTS-----YGYYNN-SIVLP-QDAK-VDEIKAEMKD--GVLTITI

At1G52560 DEYWSSKS-----YGYYNT-SLSLP-DDAK-VEDIKAELKN--GVLNLVI

Sl-SGN-U314000 DKHSWGRN-----YSSYDT-RLSLP-DNVV-KDKIKAELKN--GVLFISI

Sl-SGN-U334715 DKPSGGG------FVAGRA-LFQVS-QLMW-GGENQSGHDE--WSSFLFD

St-SGN-U274067 DEHSWGRN-----YSSYDT-RLSLP-DNVE-KDKIKAELKN--GVLFISI

At4G27670 SWSGSGRS-----VSSYGT-RLQLP-DNCE-KDKIKAELKN--GVLFITI

Sl-SGN-U339823 EEEWSAKS-----YGKYNT-RIALP-ENID-FEKIKAEVKD--GVLYITI

St-SGN-U276891 EEEWSAKS-----YGKYNT-RIALP-ENID-FEKIKAEVKD--GVLYITI

Sl-SGN-U339822 EEEWSAKS-----YGKYNT-RIALP-ENID-FEKIKAEVKD--GVLYITI

St-SGN-U271467 ------------YRRRYST-RLEIPQNIYK-LDGIKAEMKN--GVLKVAV

St-SGN-U288169 ------------YRRRYST-RLETPQNMYK-LDGIKAEMKN--GVLKVAV

Sl-SGN-U312456 ------------YRRRYST-RLEIPQNIYK-LDGIKAEMKN--GVLKVAV

Ca-SGN-U196718 ------------YRRRYST-RLEIPQKLYK-LDGIKAEMKN--GVLKVAV

At4G25200 -----------NRNRRFTS-RIGLPDKIYK-IDEIKAEMKN--GVLKVVI

At5G51440 -------------GRRFTS-RIELPEKVYK-TDEIKAEMKN--GVLKVVI

At2G19310 -------------DNKFMS-RFKLP-NNAL-TDQVTAWMED----EFLVV

At2G03020 SSGRFYSR-----FYSGDVAMLSTP-VDIP-SRRIKTIAKN--GVIRLLI

At4G16540 SSGRFYSR-----FYSGDGAMLSTP-VDIP-SRRIKTIAKD--GVIRLLI

Nt-shsp(Ralstonia) MKVQQLSP-----PGPFTV-SFNLPGPVDP-SLCSPRFRSD--GILEVIV

Nb-shsp(Ralstonia) MKVQQLSP-----PGPFTV-SFNLPGPVDP-RLCSRRFRSD--GILEVIV

At5G47600 NAGRAGRK------YGGS--MIFNP-EAYD-VKKTKVKLIN--GVLWITV

At1G06460 ASILGYHK-----QGPFKV-SWPLP-SNVN-KDNVSAEFMD--GIL----

At1G12180 -HSLP--------VRTYIG-FVKFP-AVYD-SKHAKIWVVN--GVLWITV

Sl-SGN-U329774 ------------SGRKYGG-SLEFS-TDCCKAEKVEAQMKN--GILRMVI

At5G47590 ------------SGRTYLG-SVQSP-FPALISNNTIAWDAEF-GVLRIAV

Sl-SGN-U326583 PR-TLV--RRGFFIEPDDLT------------------------

St-SGN-U274954 PR-TLV--RRGFFIEPDDLP------------------------

At4G21870 PK-RIM--TRR-LIDPSDVP------------------------

Sl-SGN-U329160 PW-KMP--KQLADEHTKQSA------------------------

St-SGN-U293629 PW-KMR--KQLLDEHTKQSA------------------------

Sl-SGN-U323555 PK-LIT--TSPQKKDQELPT------------------------

At5G20970 PK-LKT--EIPQTKPPTPVI------------------------

At2G27140 PRTEPM--GKQPSPIGTATK------------------------

At5G04890 PK-ETI--TKVAYLPETSRT------------------------

Sl-SGN-U340504 PK-ELPENNEREEAAIASSG------------------------

Sl-SGN-U312454 PK-EEV--KKPEVKSIEISG------------------------

St-SGN-U269409 PK-EEV--KKPEVKSIDISG------------------------

Sl-SGN-U312453 PK-EEV--KKPEVKSIEISG------------------------

Nb-DQ275464 PK-EEE--KKPDVKSIEIT-------------------------

Sl-SGN-U333698 PK-EEV--KKPEVKSMENSG------------------------

Sl-SGN-U312455 PK-EEV--KKPDVKSIEISG------------------------

St-SGN-U269408 PK-EEV--KKPDVKSIEISG------------------------

Ca-SGN-U196343 PK-AEV--KKPDVKSIEISG------------------------

Ca-SGN-U196341 PK-EEV--KNPDXX------------------------------

St-SGN-U269410 PK-EEV--KKSDVKSIDISG------------------------

Sl-SGN-U312450 PK-EEV--KKPDVKSIEISG------------------------

Ca-SGN-U196342 PK-EEV--KKPDVKSIEISG------------------------

Sl-SGN-U333697 PK-EEV--KKPEVKSIEISG------------------------

St-SGN-U287373 PK-EQV--KKPDVKSIEISG------------------------

At1G59860 PK-VETNKKKAQVKSIDISG------------------------

At1G07400 PK-VEEAKKKAQVKSIDISG------------------------

At2G29500 PK-AET--KKADVKSIQISG------------------------

At3G46230 PK-VQE--SKPEVKSVDISG------------------------

At1G53540 PK-VPE--KKPEVKSIDISG------------------------

At5G59720 PK-APE--KKPQVKSIDISG------------------------

St-SGN-U269225 PK-EEG--KKPEVKAIDISG------------------------

St-SGN-U269224 PK-EEE--KKPEVKAINISG------------------------

Sl-SGN-U316206 PK-EEE-KKKSEVKAIDISG------------------------

Sl-SGN-U316208 PK-EEE-KKKPEVKAIDISG------------------------

Sl-SGN-U316205 PK-EEE-KKKSEAKAIDISG------------------------

Sl-SGN-U316207 PK-EEE-KKKPEVKAIEISG------------------------

St-SGN-U269763 PK-EEE-KKKSEVKAIDISD------------------------

Sl-SGN-U342568 PK-EEV--KKADVKAIEISG------------------------

St-SGN-U269765 PK-EEE-KKKSEVKAIDISG------------------------

St-SGN-U269764 PK-EEE-KKKSEVKAIDISG------------------------

St-SGN-U269761 PK-EEG-KKKFGVKAIDIFG------------------------

St-SGN-U269762 PK-EEE-KKKSEVKAIDISG------------------------

Os03g16020 PK-EEP--KKPDVKSIQITG------------------------

Os02g52150 PK-VKE--EQRRDVFQVNVE------------------------

Os06g11610 PK-LKE--EERKDVFQVNVE------------------------

Os01g04370 PK-AEV--KKPEVKAIEISG------------------------

Os01g04380 PK-AEV--KKPEVKAIEISG------------------------

Os03g16030 PK-EEP--KKPDVKSIQVTG------------------------

Os03g16040 PK-EEP--KKPDVKSIQISG------------------------

Os03g15960 PK-EEA--KKPDVKSIQISG------------------------

Os01g04340 PK-EET--KKPQLKAIPISG------------------------

Os01g04350 PK-VVTD-KQPAIAAAAPVP------------------------

Os02g03570 PK-DND--RKP-HARIIPIT------------------------

Os04g36750 RKLAPDQIKGPRVVGIASAG------------------------

Os11g13980 PK-VPGHRGREPRVVAIDGA------------------------

Os06g14240 PK-EPAPARPRTRPIAVSSK------------------------

Os01g08860 EK--LPPPEPKKPKTIEVKV------------------------

Os02g54140 KK--RPPPEKKTKSV-QVTI------------------------

Os02g12610 DK-KPPP-EPKKPRVVEVKV------------------------

Sl-SGN-U333694 PK-EEV--KKPEVKSLEIFG------------------------

Sl-SGN-U333693 PK-EGG--EEPGANSMEFSG------------------------

Ca-SGN-U197520 PK-EEE--KKPEVKAIDISG------------------------

St-SGN-U273600 PK-EEV--KKPFSKLIEIED------------------------

St-SGN-U288775 PK-EQV--KKPFSKLIEIEQ------------------------

Sl-SGN-U321313 PK-DATPKTSK-VRNINITS------------------------

St-SGN-U298363 PK-DATPKTSK-VRNINITS------------------------

At5G37670 PK-DTSSKSSK-VRNVNITS------------------------

Sl-SGN-U325739 PK-KETQQVPKNVKAINIT-------------------------

Sl-SGN-U332227 PK--LAEEKKKQSKVISIAE------------------------

Sl-SGN-U341636 PK--LAEEEKKQSKVISISE------------------------

Sl-SGN-U318540 AK--LSADRIKGPKVVSIES------------------------

St-SGN-U281516 AK--LSADRIKGPKVVSIES------------------------

At4G10250 TK--LSPEKVKGPRVVNIAA------------------------

Sl-SGN-U332716 EK--MPP--PSKAKTVKVAV------------------------

At1G54050 KK--LPPQ-PPKPKTVQIAV------------------------

At5G12020 QK--LPPPEPKKPKTIQVQV------------------------

At5G12030 PK--LPPPEPKKPKTIQVQV------------------------

Sl-SGN-U316987 QK--LPPPEPKKPKTIEVKV------------------------

St-SGN-U271074 QK--LPPPEPKKPKTIEVKV------------------------

Sl-SGN-U316986 QK--LPPPEPKKSKTIQVKV------------------------

Sl-SGN-U335906 QK--LPPPEPKKPKTIQVKV------------------------

Ca-SGN-U196893 HK--LPPPEPKKPKTIEVKV------------------------

St-SGN-U271057 PK-KEIRKNQHHLRSVQIN-------------------------

Sl-SGN-U321049 PK-KEISRNHHHIRSVQIN-------------------------

Ca-SGN-U207893 PK-KEIKKP--HLRTVEIH-------------------------

Sl-SGN-U321004 PK-KGAVKKS-HVRTVRIF-------------------------

St-SGN-U298302 PK-KGAKKSRTFFSNTILKY------------------------

St-SGN-U273896 PK-SEKPKK--DVKEIQVM-------------------------

Sl-SGN-U321169 PK-SDKPKK--DVKEIEVL-------------------------

At1G52560 PR-TEKPKK--NVQEISVE-------------------------

Sl-SGN-U314000 PK-TEVEKK-VIDVQINNI-------------------------

Sl-SGN-U334715 SK-VEVGKK-VEMSKLNDI-------------------------

St-SGN-U274067 PK-TKVEKK---VVDVQIN-------------------------

At4G27670 PK-TKVERK---VIDVQIQ-------------------------

Sl-SGN-U339823 PK-ASSNPK---VFDINFN-------------------------

St-SGN-U276891 PK-ASSNPK---VFDINVQ-------------------------

Sl-SGN-U339822 PK-ASSNPK---VFDINVQW------------------------

St-SGN-U271467 PK-VKQEERK-DVFDVKIE-------------------------

St-SGN-U288169 PK-VKQEERK-DVFDVKIE-------------------------

Sl-SGN-U312456 PK-VKQEERK-DVFDVKIE-------------------------

Ca-SGN-U196718 PK-VKEEERK-DVFNVQVE-------------------------

At4G25200 PK-MKEQERN-DVRQIEIN-------------------------

At5G51440 PK-IKEDERN-NIRHINVD-------------------------

At2G19310 FV--EKDASSSPPQLPEIEE------------------------

At2G03020 PP-F----------------------------------------

At4G16540 PP-F----------------------------------------

Nt-shsp(Ralstonia) LK-YRIPIVSAEGLPENWYNGSFPAP------------------

Nb-shsp(Ralstonia) LK-YRIPIVSAEGLPENWCNGSFPAP------------------

At5G47600 PK-IPGKNASINVKERIL--------------------------

At1G06460 --------------------------------------------

At1G12180 TK-HQGRQG-----------------------------------

Sl-SGN-U329774 KG-EMGEDX-----------------------------------

At5G47590 IP-PDDMTTINNKRNPIE--------------------------
