# Supplementary material for: The small heat shock protein 20 RSI2 interacts with and is required for stability and function of tomato resistance protein I-2
Source: Plant J. 2010 Jun 16;63(4):563–72. doi: 10.1111/j.1365-313X.2010.04260.x (PMC2988412; doi:10.1111/j.1365-313X.2010.04260.x)
Supplement: Supplementary file 1 [file tpj0063-0563-SD1.pdf]

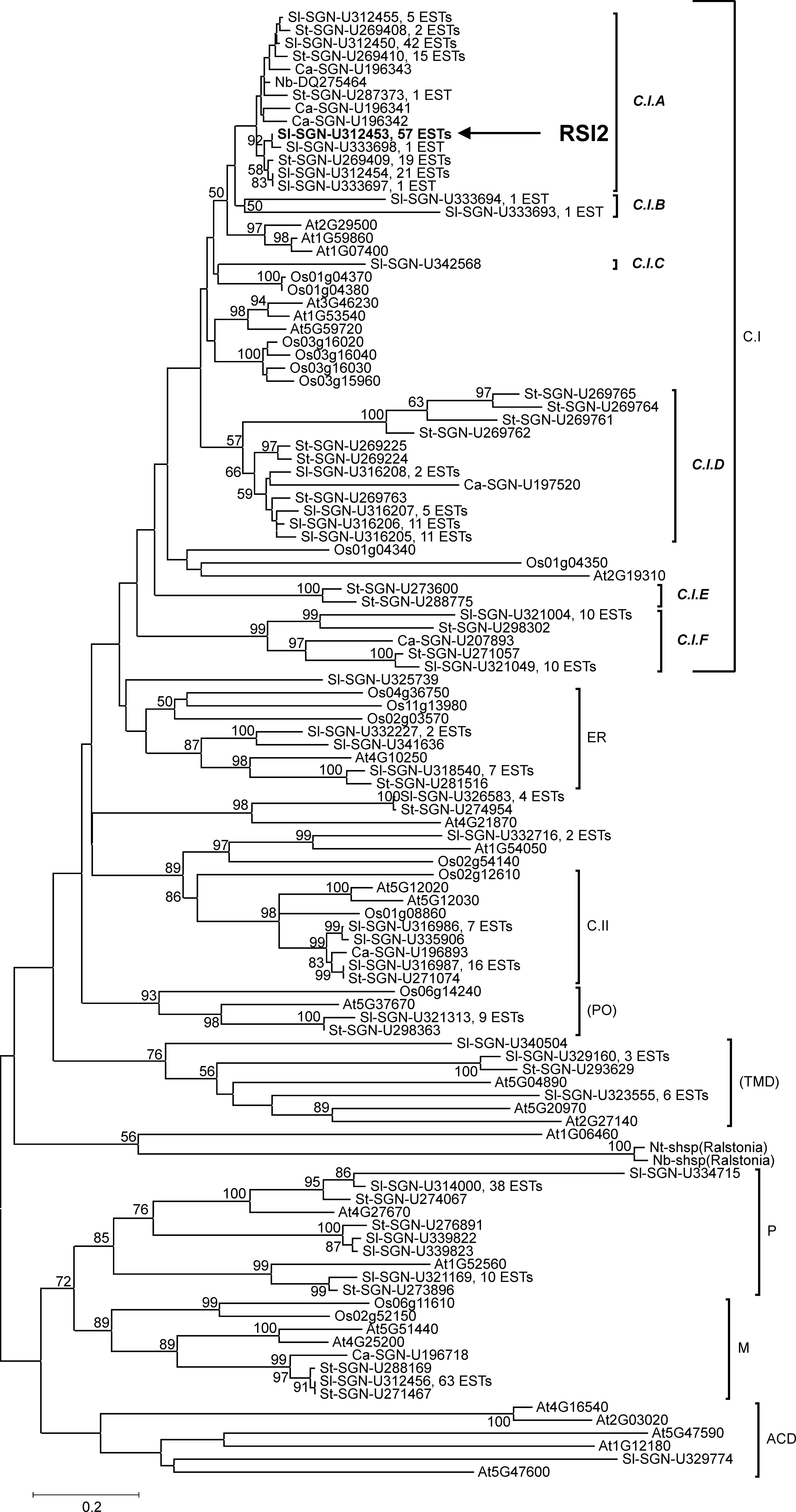

Sl-SGN-U312455, 5 ESTs
St-SGN-U269408, 2 ESTs
Sl-SGN-U312450, 42 ESTs
St-SGN-U269410, 15 ESTs
Ca-SGN-U196343
Nb-DQ275464
St-SGN-U287373, 1 EST
Ca-SGN-U196341
Ca-SGN-U196342
92
Sl-SGN-U312453, 57 ESTs
RSI2
Sl-SGN-U333698, 1 EST
St-SGN-U269409, 19 ESTs
58
Sl-SGN-U312454, 21 ESTs
83
Sl-SGN-U333697, 1 EST
C.I.A
50
Sl-SGN-U333694, 1 EST
50
Sl-SGN-U333693, 1 EST
C.I.B
97
At2G29500
98
At1G59860
At1G07400
Sl-SGN-U342568
C.I.C
100
Os01g04370
Os01g04380
94
At3G46230
98
At1G53540
At5G59720
Os03g16020
100
Os03g16040
Os03g16030
Os03g15960
C.I
97
St-SGN-U269765
63
St-SGN-U269764
100
St-SGN-U269761
St-SGN-U269762
57
97
St-SGN-U269225
St-SGN-U269224
66
Sl-SGN-U316208, 2 ESTs
Ca-SGN-U197520
59
St-SGN-U269763
Sl-SGN-U316207, 5 ESTs
Sl-SGN-U316206, 11 ESTs
Sl-SGN-U316205, 11 ESTs
C.I.D
Os01g04340
Os01g04350
At2G19310
100
St-SGN-U273600
St-SGN-U288775
C.I.E
99
Sl-SGN-U321004, 10 ESTs
99
St-SGN-U298302
97
Ca-SGN-U207893
100
St-SGN-U271057
Sl-SGN-U321049, 10 ESTs
C.I.F
Sl-SGN-U325739
50
Os04g36750
Os11g13980
Os02g03570
100
Sl-SGN-U332227, 2 ESTs
87
Sl-SGN-U341636
98
At4G10250
100
Sl-SGN-U318540, 7 ESTs
St-SGN-U281516
ER
98
100Sl-SGN-U326583, 4 ESTs
St-SGN-U274954
At4G21870
99
Sl-SGN-U332716, 2 ESTs
97
At1G54050
89
Os02g54140
Os02g12610
100
At5G12020
86
At5G12030
98
Os01g08860
99
Sl-SGN-U316986, 7 ESTs
99
Sl-SGN-U335906
Ca-SGN-U196893
83
Sl-SGN-U316987, 16 ESTs
99
St-SGN-U271074
C.II
93
Os06g14240
At5G37670
100
Sl-SGN-U321313, 9 ESTs
98
St-SGN-U298363
(PO)
76
Sl-SGN-U340504
Sl-SGN-U329160, 3 ESTs
56
St-SGN-U293629
100
At5G04890
Sl-SGN-U323555, 6 ESTs
89
At5G20970
At2G27140
(TMD)
56
At1G06460
100
Nt-shsp(Ralstonia)
Nb-shsp(Ralstonia)
86
Sl-SGN-U334715
95
Sl-SGN-U314000, 38 ESTs
100
St-SGN-U274067
76
At4G27670
100
St-SGN-U276891
85
Sl-SGN-U339822
87
Sl-SGN-U339823
99
At1G52560
Sl-SGN-U321169, 10 ESTs
99
St-SGN-U273896
P
72
99
Os06g11610
Os02g52150
89
100
At5G51440
At4G25200
89
Ca-SGN-U196718
99
St-SGN-U288169
97
Sl-SGN-U312456, 63 ESTs
91
St-SGN-U271467
M
At4G16540
100
At2G03020
At5G47590
At1G12180
Sl-SGN-U329774
At5G47600
ACD
0.2
